# Enrichment of CD146^+^ Adipose-Derived Stem Cells in Combination with Articular Cartilage Extracellular Matrix Scaffold Promotes Cartilage Regeneration

**DOI:** 10.7150/thno.33904

**Published:** 2019-07-09

**Authors:** Xu Li, Weimin Guo, Kangkang Zha, Xiaoguang Jing, Mingjie Wang, Yu Zhang, Chunxiang Hao, Shuang Gao, Mingxue Chen, Zhiguo Yuan, Zhenyong Wang, Xueliang Zhang, Shi Shen, Haojiang Li, Bin Zhang, Hai Xian, Yuan Zhang, Xiang Sui, Ling Qin, Jiang Peng, Shuyun Liu, Shibi Lu, Quanyi Guo

**Affiliations:** 1 Institute of Orthopedics, Chinese PLA General Hospital; Beijing Key Lab of Regenerative Medicine in Orthopaedics; Key Laboratory of Musculoskeletal Trauma & War Injuries,PLA; 28 Fuxing Road, Haidian District, Beijing 100853, China.; 2 School of Medicine, Nankai University, Tianjin, 300071, China; 3 Musculoskeletal Research Laboratory, Department of Orthopaedics and Traumatology, Innovative Orthopaedic Biomaterial and Drug Translational Research Laboratory, Li Ka Shing Institute of Health Sciences, The Chinese University of Hong Kong, Hong Kong, China; 4 Department of Orthopaedic Surgery, First Affiliated Hospital, Sun Yat-sen University, Guangzhou, Guangdong, China; 5 Center for Biomedical Material and Tissue Engineering, Academy for Advanced Interdisciplinary Studies, Peking University, Beijing 100871, China

**Keywords:** CD146, cartilage tissue engineering, mesenchymal stem cell, scaffold, extracellular matrix

## Abstract

Heterogeneity of mesenchymal stem cells (MSCs) influences the cell therapy outcome and the application in tissue engineering. Also, the application of subpopulations of MSCs in cartilage regeneration remains poorly characterized. CD146+ MSCs are identified as the natural ancestors of MSCs and the expression of CD146 are indicative of greater pluripotency and self-renewal potential. Here, we sorted a CD146^+^ subpopulation from adipose-derived mesenchymal stem cells (ADSCs) for cartilage regeneration.

**Methods**: CD146^+^ ADSCs were sorted using magnetic activated cell sorting (MACS). Cell surface markers, viability, apoptosis and proliferation were evaluated *in vitro*. The molecular signatures were analyzed by mRNA and protein expression profiling. By intra-articular injections of cells in a rat osteochondral defect model, we assessed the role of the specific subpopulation in cartilage microenvironment. Finally, CD146^+^ ADSCs were combined with articular cartilage extracellular matrix (ACECM) scaffold for long term (3, 6 months) cartilage repair.

**Results**: The enriched CD146^+^ ADSCs showed a high expression of stem cell and pericyte markers, good viability, and immune characteristics to avoid allogeneic rejection. Gene and protein expression profiles revealed that the CD146^+^ ADSCs had different cellular functions especially in regulation inflammation. In a rat model, CD146^+^ ADSCs showed a better inflammation-modulating property in the early stage of intra-articular injections. Importantly, CD146^+^ ADSCs exhibited good biocompatibility with the ACECM scaffold and the CD146^+^ cell-scaffold composites produced less subcutaneous inflammation. The combination of CD146^+^ ADSCs with ACECM scaffold can promote better cartilage regeneration in the long term.

**Conclusion**: Our data elucidated the function of the CD146^+^ ADSC subpopulation, established their role in promoting cartilage repair, and highlighted the significance of cell subpopulations as a novel therapeutic for cartilage regeneration.

## Introduction

Articular cartilage, lacking blood vessels, lymphatic vessels, and nerves, is not subjected to systemic regulation 1. Cartilage is susceptible to damage, and acute lesions without proper treatment may result in post-traumatic osteoarthritic progression 2, 3. Traditionally, bone marrow stimulating procedures, including abrasion, subchondral drilling, and microfracture techniques, can mobilize progenitor cells from the subchondral bone to promote cartilage repair 4-6. However, hyaline repair tissue cannot be consistently induced, and most often it does not suffice to fill the entirety of the defect. Currently, autologous chondrocyte implantation is used in clinical practice 7, but issues of limited availability, de-differentiation, and functional loss during *in vitro* culturing limit its application 8. Stem cells, because of their high cell viability, and multilineage differentiation capacity, are therefore being investigated as alternative cell sources for cartilage regeneration 9-12.

Research on stem cell-based tissue engineering has until now mostly concentrated on harvesting mesenchymal stem cells (MSCs) from different sources, such as bone marrow 13, 14, adipose tissue 15, synovium 16, the bloodstream 17, umbilical cord/Wharton's jelly 18, amniotic fluid 19 and the amnion 20, for exploring their self-renewal and differentiation capacities and their application in *in vivo* cartilage repair. However, MSCs are a heterogeneous population, which cannot be identified and isolated using single surface markers 21, 22, and therefore MSC-based clinical trial outcomes vary 21. Hence, the cellular heterogeneity of MSCs affects the treatment outcome. In this context, the use of specific subpopulations of MSCs in tissue regeneration is an emerging idea and represents an innovative approach.

CD146, also known as MUC18, MCAM, Mel-CAM, or S-Endo1, is a transmembrane glycoprotein and an adhesion molecule of the immunoglobulin (Ig) superfamily 23, 24. CD146 was initially known as an endothelial biomarker and a marker for melanoma progression 25. Later, it was also recognized as a marker for pericytes 24, 26, which have been identified as the natural ancestors of MSCs 27, 28. In human tissues, CD146^+^ cell was regarded as the mural cells of blood micro vessels 29, 30. Previous reports also showed that CD146 was associated with cell migration and self-renewal 31, 32. Hagmann et al. found that CD146^+^ bone marrow MSCs (BMSCs) increased GAG/DNA content after enrichment 33. Wu et al. reported that a CD146^+^ subpopulation from human umbilical cord cells could treat arthritis and provide an anti-inflammatory protective microenvironment by suppressing IL-6 34. Su et al. showed that CD146^+^ chondroprogenitors expressed higher levels of an MSC-specific marker and had better chondrogenic differentiation capacity 35. Thus, we hypothesized that CD146^+^ subpopulations have the potential to promote better cartilage repair.

For clinical applications, sorting CD146^+^ cells from adipose-derived MSCs (ADSCs) has many advantages. ADSCs are maintainable *in vitro* for longer times than BMSCs and exhibit stable population doubling with higher proliferation and lower senescence rates. Fewer ADSC passages are required to obtain adequate cell numbers. hADSCs can be derived from lipoaspirate, which can be harvested through tumescent abdominal liposuction techniques. A sufficient number of cells can be obtained in one attempt which avoids the morbidity associated with harvest 10, 36. A recent study compared the differences of ADSCs and BMSCs at single- and bulk-cell levels on the treatment of osteoarthritis, they found ADSCs as a more stable and controllable stem cell source, was more adaptable to surviving in the hypoxic articular cavity niche, and exhibited superiority in regulating inflammation 37.

In this study, we report a CD146^+^ MSC subpopulation-based cartilage tissue engineering strategy. The CD146^+^ subpopulation was enriched from human ADSCs. Cellular characteristics, including the expression of the surface marker, immune character, viability, proliferation gene and cytokine expression patterns were identified *in vitro*. Further, the role of the CD146^+^ subpopulation in the cartilage microenvironment was explored *in vivo*. To provide a more suitable microenvironment for cell transplantation, we also developed an articular cartilage extracellular matrix (ACECM) cell-scaffold composite and investigated its biocompatibility and its role in repairing cartilage defects. We successfully identified and isolated CD146^+^ subpopulations from hADSCs, investigated their cytological functions, and established their role in cartilage regeneration.

## Methods

### hADSC culture

Human adipose-derived mesenchymal stem cells (hADSCs) at passage 4 were provided by the National Cell Bank for Tissue Engineering (Zhejiang, China). hADSCs were plated in growth medium consisting of Dulbecco's Modified Eagle Medium/ Fisher12 (DMEM/F12) medium (10% fetal bovine serum, 1% penicillin-streptomycin added), followed by incubation in a humidified incubator at a temperature of 37 °C. Cells were harvested when they reached ~80% confluence using 0.25% trypsin in ethylenediaminetetraacetic (EDTA; Invitrogen).

### Isolation of CD146+ cells from hADSCs

**Figure 1:**
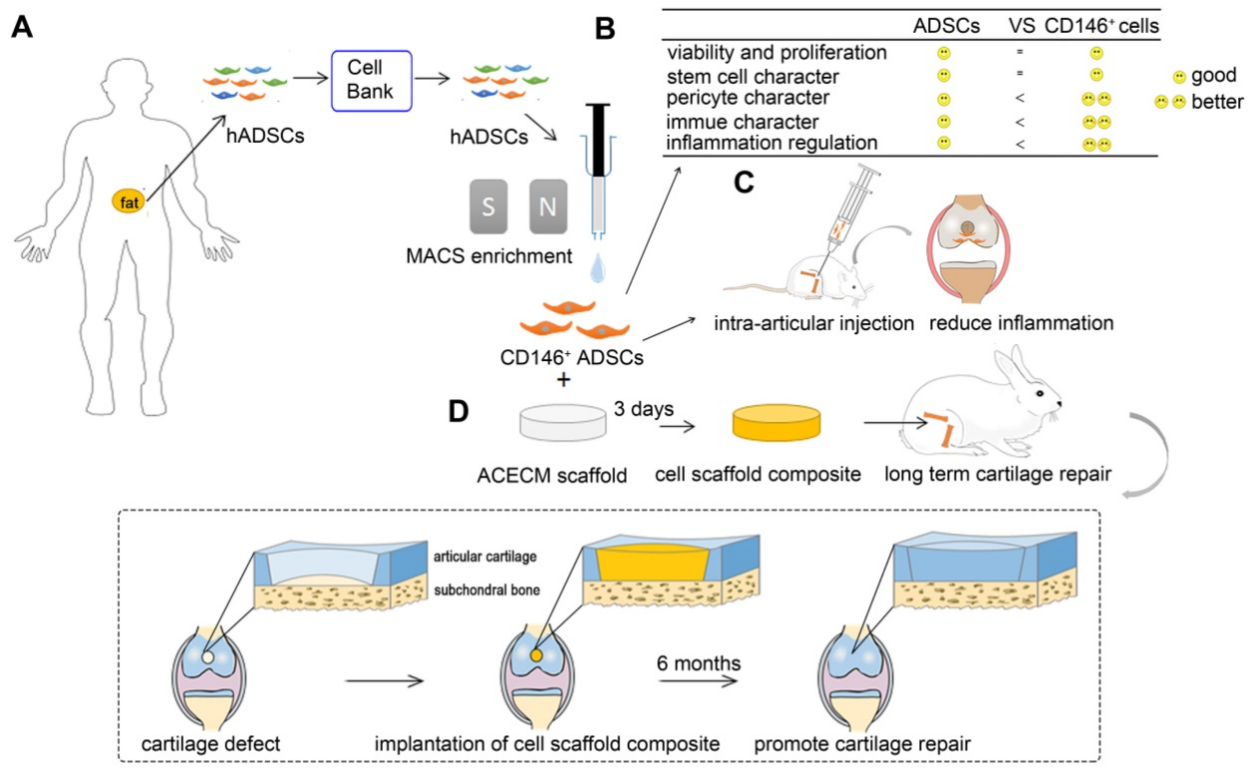
** Overview of experimental design.** (A) MACS enrichment of CD146^+^ ADSCs. (B) Comparison of cytological characteristics between ADSCs and CD146^+^ ADSCs *in vitro*. CD146^+^ ADSCs retained good viability, proliferation ability, and stem cell character after sorting. CD146^+^ ADSCs also showed pericyte characteristics, better immune properties, and inflammation regulation ability. (C) A schematic illustration of the intra-articular injection of CD146^+^ ADSCs in rats. (D) Flow chart of the preparation of the cell-scaffold composite for long term cartilage repair in rabbits.

CD146+ cells were isolated from a suspension of passage 5 hADSCs by magnetic activated cell sorting (MACS) using anti-CD146 magnetically labeled antibodies (catalog 130-093-596, Miltenyi Biotec) (Figure 1A).

### Flow cytometry

A 100-mL aliquot of hADSCs or freshly sorted CD146^+^ cells suspension (1×10^6^/mL) was transferred to the test pipe. Cells were stained with anti-human antibodies against the following cell surface markers: CD34-PE (BD Pharmingen), CD45-FITC (BD Pharmingen), CD73-PE (BD Pharmingen), CD90-FITC (BD Pharmingen), CD105-PE (BD Pharmingen), CD146-PE (BD Pharmingen), and HLA-DR-FITC (BD Pharmingen). The cells were incubated in the dark for 30 min, collected on a FACS Calibur flow cytometer, and the results were analyzed using Cell-Quest for Macintosh Software.

### Western blotting

Total proteins were extracted from hADSCs and freshly sorted CD146^+^ cells using RIPA buffer with PMSF. After quantifying the protein concentration using a BCA protein assay kit, proteins were subjected to SDS-PAGE and transferred to a PVDF membrane. The membrane was incubated with primary antibodies against the following proteins: HLA-1 (1:1000; Abcam), MHC-II (1:10,000; Abcam), CD40 (1:500; Abcam), CD80 (1:500; Abcam), CD86 (1:5000; Abcam), NG2 (1:5000; Novus Biologicals), PDGFR-β (1:1000; Abcam), RGS5 (1:1000; Novus Biologicals), and GAPDH (1:10,000). Subsequently, the membrane was washed with Tris-buffered saline (TBS) containing 0.05% Tween-20 and incubated with an anti-mouse or an anti-rabbit HRP-conjugated secondary antibody. The antigen-antibody reaction was visualized using enhanced chemiluminescence assay (Western Luminescent Detection Kit) and documented using Gel Doc (Bio-Rad).

### Immunocytochemical and immunofluorescence staining

For immunocytochemical staining, cells were fixed with 4% paraformaldehyde (PFA) for 20 min at room temperature, treated with methanol for 10 min, washed with PBS, permeabilized with 0.25% Triton X-100 in PBS for 20 min, and blocked with 5% goat serum in 0.25% Triton X-100 in PBS for 20 min. Next, cells were incubated with anti-HLA Class I (Abcam, ref. ab23755) and anti-MHC-II (Abcam, ref. ab157210) at 4°C overnight. The immunodetection was performed using DAB (MXB biotechnologies), following the manufacturer's instructions. Cells were viewed with an Olympus BX51 light microscope (Olympus, Tokyo, Japan). The images were captured using an Olympus CCD DP71 (Olympus).

For immunofluorescence staining, cells were fixed with 4% PFA for 20 min at room temperature, treated with methanol for 10 min, washed with PBS, permeabilized with 0.25% Triton X-100 in PBS for 20 min, and blocked with 5% goat serum in 0.25% Triton X-100 in PBS for 20 min. Subsequently, cells were incubated with anti-CD146 (Abcam), anti-PDGFR-β (Abcam), anti-RGS5 (Novus Biologicals), or anti-NG2/MCSP (Novus Biologicals) at 4°C overnight. On the the next day, cells were washed and incubated with secondary antibody in 5% goat serum for 1 h at room temperature. Cells were counterstained with a 1:1000 dilution of DAPI to label the nuclei and viewed with an Olympus BX51 light microscope (Olympus, Tokyo, Japan). The images were captured by using an Olympus CCD DP71 (Olympus).

### Quantitative real-time PCR

Total RNA from hADSCs and freshly sorted CD146^+^ cells was isolated using Trizol following the manufacturer's instructions (Sigma (T9424)). The cDNA was synthesized using the SuperScript TM III First-strand synthesis system for RT-PCR, cat. No: 18080-051 (Invitrogen). Quantitative RT-PCR was performed using the Power SYBRgreen PCR Master Mix (ABI, P/N: 4367659). Primers were shown in Table S1. The PCR cycling condition included an initial denaturation at 95°C for 2 min, 40 cycles of 95°C for 15 s, 58°C for 15 s, and 72°C for 35 s, and a final extension at 72°C for 5 min. The results were analyzed using Bio-Rad iQ5 optical system software.

### Cell proliferation evaluation by Cell Counting Kit-8 (CCK-8)

Cells were seeded at 3000 cells per well in a 96-well plate and cultured in a humidified incubator at a temperature of 37°C. The relative cell number was determined using CCK8 by OD (absorbance) value after 1, 3, and 5 days of culturing, following the manufacturer's instructions. The morphology of cells on was observed by phase-contrast microscopy (Olympus, BH-2).

### Apoptosis and viability analysis

The hADSCs and freshly sorted CD146^+^ cells were trypsinized using 0.25% trypsin-EDTA and harvested by centrifugation. Apoptosis of ADSCs and CD146+ cells was detected using Muse Count & Viability Kit (MCH100102). Briefly, cells were incubated with Muse Annexin V & Dead Cell reagent for 20 min at room temperature as per with the manufacturer's instructions and analyzed using Merck&Millipore Muse Cell Analyzer. Viability of ADSCs and CD146^+^ cells was determined using Muse Annexin V & Dead Cell Kit (MCH100105). Cell suspension and Muse Count & Viability reagent were added to each tube, incubated for 5 min at room temperature, and analyzed using the Merck& Millipore Muse Cell Analyzer.

### Microarray analyses

Total RNA was isolated from hADSCs and freshly sorted CD146^+^ cells using Trizol reagent and purified with mirVana mRNA Isolation Kit (Ambion, Austin, TX, USA). The RNA integrity was determined using the RNA 6000 Nano Lab-on-a-Chip kit and the Bioanalyzer 2100 (Agilent Technologies, Santa Clara, CA, USA). Only RNA extracts with RNA integrity number values >6 were used for further analysis. Gene expression analyses were performed using the Agilent human mRNA Array. The array data were analyzed with the help of Capitalbiotech for data summarization, normalization, and quality control by using the GeneSpring software V13 (Agilent). To select the differentially expressed genes, we used threshold values of ≥2- and ≤0.5-fold change and a Benjamini-Hochberg-corrected *P*-value of 0.05. The data were log(2)-transformed and median-centered by genes using the Adjust Data function of CLUSTER 3.0 software. The data were further analyzed with hierarchical clustering with average linkage 38. Tree visualization was performed by using Java Treeview (Stanford University School of Medicine, Stanford, CA, USA). Gene ontology (GO) pathway enrichment analysis was performed by WEGO (http://wego.genomics.org.cn/cgi-bin/wego/index.pl).

### Cytokine analysis

The ADSCs and CD146^+^ cells were lysed and quantified. The lysates were analyzed for chemokines and growth factors (80 cytokines in total) using a RayBio® Human Cytokine Antibody Array G5 (RayBiotech, USA), following the manufacturer's instructions. Data were analyzed using GenePix Pro 6.0.

### Rat osteochondral defect model and histological examination

Eight-week-old SD rats with a mean weight of 220 ± 20 g were used in this study (Figure 1C). Using an electric drill, osteochondral defects (1.5 mm diameter and 1 mm depth) were created through both the chondral and the subchondral bone layer of the patellar trochlear groove in their right legs. The joint capsule and the overlying muscle were closed with a suture. All rats were randomly allocated into four groups: defects with intra-articular injections of PBS (negative control), defects with intra-articular injections of ADSCs (1×10^5^), defects with intra-articular injections of CD146^+^ cells (1×10^5^), and the sham-operated group. The knee joint samples were collected 2 weeks after the operation. For histological examination, the samples were stained with immunohistochemistry staining of HLA-ABC (Abcam, 1:100), IL-1β (Abcam, 1:100), IL-6 (Abcam, 1:100), IL-10 (Abcam, 1:100), H&E, Safranine O, and Toluidine Blue.

### Multiplex cytokine assay

Areas of the cartilage and subchondral bone with defects were selected. Inflammatory cytokines present in the cartilage and subchondral bone samples were detected following the manufacturer's instructions (RayBiotech, USA). Data were analyzed using GenePix Pro 6.0.

### Fabrication of cell-scaffold composites

The synthesis of the Production of the ACECM scaffold was described previously 39. The diameter of the scaffolds was 3.5 mm and the height of the scaffold was 2 mm. A total number of 5×10^5^ hADSCs or freshly sorted CD146^+^ cells were seeded into ACECM scaffolds and cultured in DMEM/F12 for 3 days.

### Scanning electron microscopy

After being in cluture for 3 days, the microarchitecture of cell-scaffold composites was analyzed using the scanning electron microscopy (SEM) (S-2600N, Japan). Cell-scaffold composites, after de-hydrating in a graded ethanol series, were dried in a critical point dryer and coated with gold-palladium (EMS850X, USA).

### Live/dead staining and confocal microscopy for cell viability/growth

After being in culture for 3 days, the viability and growth of cells in the scaffold were assessed by live/dead staining following the manufacturer's guidelines (Sigma). The viable cells were labeled in green and dead cells were labeled in red. Live and dead cells were visualized and scored using fluorescence imaging and confocal microscopy (Leica Microsystems DM6000B-SP57CS).

### *In vivo* biocompatibility and degradability

Cell-scaffold composites were subcutaneously embedded into the back skin of New Zealand white rabbits (~2.0 kg) to evaluate their degradation and biocompatibility. At 1, 2, and 4 weeks after implantation, rabbits were euthanized, and photographs of the remaining cell-scaffold composites under the skin were taken. H&E staining was also carried out to evaluate the histological changes. Immunofluorescence staining of HLA-ABC (Abcam) was performed to evaluate human cell persistence overtime.

### *In vivo* surgical procedure of full-thickness rabbit cartilage defect repair

Sixty healthy New Zealand white rabbits (~2.0 kg) were employed for the *in vivo* cartilage defect repair experiments (Figure 1D). The region of interest was shaved and aseptically prepared for operation. Using an electric drill, a cartilage defect (3.5 mm diameter and 2 mm depth) was created through the chondral layers of the patellar trochlear groove in their right legs. The defects of the experimental group were implanted with ACECM scaffold, ADSCs & ACECM scaffold composites, and CD146^+^ subpopulation & ACECM scaffold composites. The defects of the negative control group received no treatment. A sham-operated group was also included. Each group consisted of 12 healthy New Zealand white rabbits. Animals were housed individually and given penicillin for 7 days following the operation. All rabbits were euthanized at the pre-determined time points (3 or 6 months). The knee joints were collected for further evaluation.

### X-ray and magnetic resonance imaging

The samples were first assessed using an X-ray scanner (Faxitron, USA). Subsequently, the magnetic resonance imaging (MRI) scans were taken using a 7.0 T Bruker Biospec system (Bruker Biospec, Germany). T2-weighted spin-echo images with fat suppression were obtained in the sagittal, frontal, and transverse planes. All images were scored independently following the Score System for MRI Evaluation 40 (Table S2).

### Macroscopic observations

The cartilage defect area in the femoral condyles and the tibial plateau was observed and photographed. All images were scored independently by an experienced researcher specialized in musculoskeletal disease and blinded to the group assignments, following the International Cartilage Repair Society (ICRS) macroscopic evaluation guidelines (Table S3).

### Histology and histomorphometry

For histological examination, tissue samples were fixed in 4% PFA, de-calcified in EDTA, and prepared for paraffin-embedded sections. The regenerated cartilage was sectioned into 6-μm slices and stained with H&E, Safranin-O, Toluidine blue and Sirius Red following the manufacturer's protocols. All images were scored independently by an experienced researcher specialized in musculoskeletal disease and blinded to the group assignments according to the ICRS histological assessment scale (Table S4).

### Statistics

All quantitative data were analyzed using SPSS version 22.0 (SPSS, Chicago, IL, USA) and reported as the mean ± standard error of the mean. Student's *t*-test or one-way analysis of variance (one-way ANOVA), followed by the Bonferroni multiple comparison test, was performed for normally distributed data. A value of *P* < 0.05 was considered to indicate a statistically significant difference.

### Study approval

All animal experiments were performed strictly following the standards for the care and use of laboratory animals and were approved by the Institutional Animal Care and Use Committee of the Chinese PLA General Hospital.

## Results

### Enriched CD146^+^ subpopulation retains the expression of mesenchymal stem cell and pericyte surface markers, and has a better immune character

**Figure 2:**
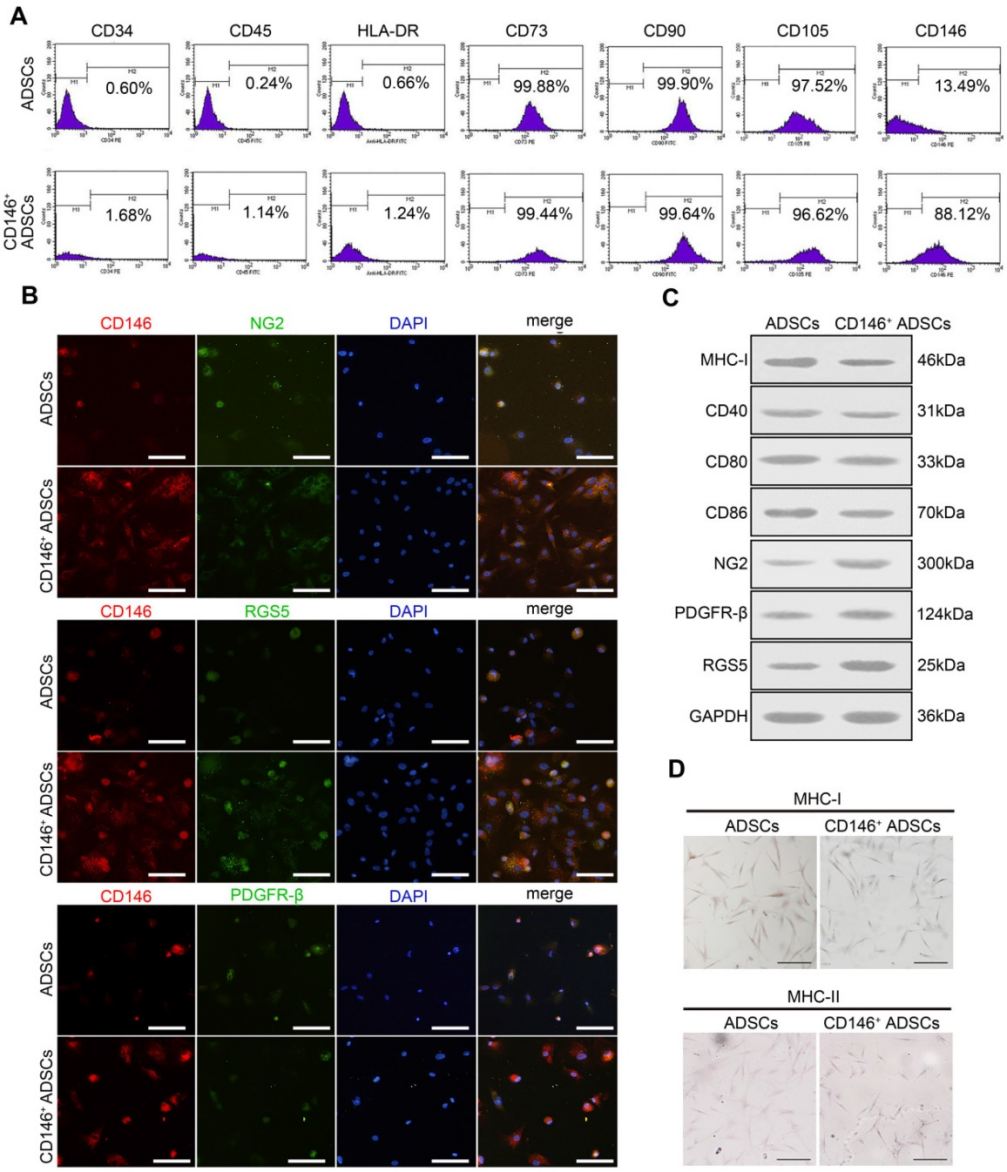
** Surface markers expression and immune characteristics of CD146^+^ ADSCs before and after MACS enrichment.** (A) Flow cytometric analysis of MSC-specific surface markers in CD146^+^ ADSCs and donor-matched ADSCs. CD146+ ADSCs and donor-matched ADSCs were stained for CD34, CD45, CD73, CD90, CD105, CD146, and HLA-DR. (B) Immunofluorescence images for CD146 (red), NG2 (green) RGS5 (green) and PDGFR-β (green) in CD146^+^ ADSCs and donor-matched ADSCs. Scale bar: 20 μm. (C) Western blots analysis of MHC-I, MHC-II, CD40, CD80, CD86, NG2, PDGFR-β, and RGS5. MHC-II was not detected in either group. (D) Representative images of MHC-I and MHC-II immunohistochemical staining in CD146^+^ ADSCs and donor-matched ADSCs. Scale bar: 50 μm.

CD146^+^ subpopulation was sorted by MACS and the expression of stem cell surface markers was examined. Similar to hADSCs, CD146^+^ subpopulation expressed the cell surface markers CD73, CD90, and CD105, but did not express CD34, CD45, and HLA-DR (Figure 2A). After the enrichment, the percentage of the CD146^+^ subpopulation increased from 13.48% to 88.12% (Figure 2A). To determine the expressions of other pericyte surface markers in CD146^+^ subpopulation, immunofluorescence staining and Western blotting were performed (Figure 2B-C). As shown by immunofluorescence staining, the CD146^+^ subpopulation co-expressed the other pericyte markers NG2, PDGFR-β, and RGS5 (Figure 2B). Western blotting demonstrated that the expression levels of these markers in CD146^+^ subpopulation were higher than those in hADSCs before enrichment (Figure 2C).

We further investigated the immune characteristics of the CD146^+^ subpopulation. The expression of MHC determines the matching of allografts with hosts. As validated by immunocytochemistry and Western blotting, the expression of MHC-I was faintly positive in CD146^+^ subpopulation, and was lower than hADSCs before sorting (Figure 2D), whereas the expression of MHC-II was negative in both ADSCs and the CD146^+^ subpopulation (Figure 2D). CD40, CD80 (B7-1), and CD86 (B7-2) are co-stimulatory molecules, which interact with CD28 of T-cells and form the second signal necessary for T-cell activation. The absence of these molecules causes T-cell anergy. No significant difference was found in the expression levels of CD40, CD80, and CD86 between the CD146^+^ subpopulation and hADSCs as determined by qPT- PCR and Western blotting (Figure 2C). IDO, PGE2, IL-10, and HGF are molecules that have an immune suppressive effect. Although the expression levels of IDO in the CD146^+^ subpopulation were lower than those in ADSCs, the expression levels of PGE2 and IL-10 were significantly higher in the CD146^+^ subpopulation (Figure S2).

### CD146^+^ subpopulation retains a high proliferation rate and viability after the enrichment

**Figure 3:**
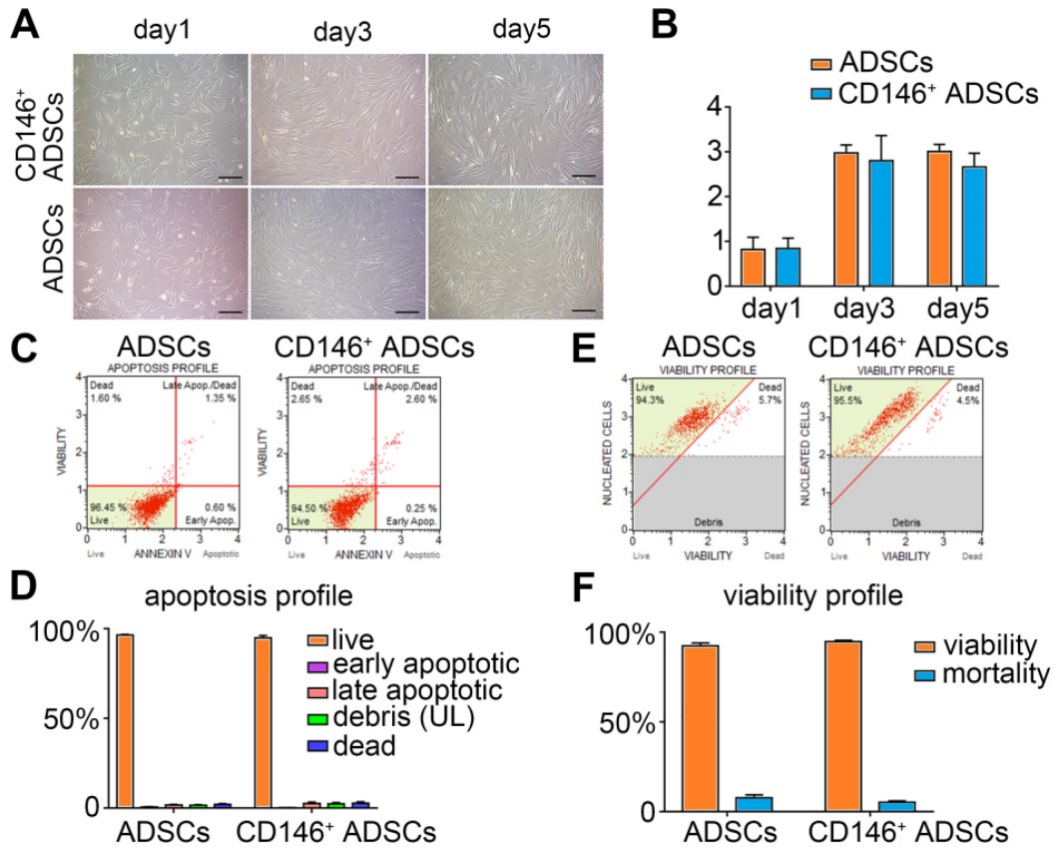
** Proliferation, apoptosis, and viability of CD146^+^ ADSCs before and after MACS enrichment.** (A) Morphology of CD146^+^ ADSCs and donor-matched ADSCs on day 1, 3, and 5. Cells were cultured at the same density on day 1. Scale bar: 50 μm. (B) Cell proliferation CCK8 assay of ADSCs and CD146^+^ ADSCs. *P < 0.05, pairwise comparisons, Student's *t*-test. (C) Apoptosis in ADSCs and CD146^+^ subpopulation was determined by flow cytometric analysis. (D) The proportion of apoptotic ADSCs and CD146^+^ subpopulation. The proportions of live, early apoptotic, late apoptotic, debris, and dead cells of the CD146^+^ subpopulation are shown in mean ± standard error, ***P < 0.05 by Student's *t*-test compared with ADSCs. (E) Viability in ADSCs and CD146^+^ cells by flow cytometry. (F) Cell viability of ADSCs and CD146^+^ subpopulation. The proportions of live and dead CD146^+^ cells are shown in the mean ± standard error of the mean, ***P < 0.05 by Student's *t*-test compared with ADSCs.

Proliferation capacity of the CD146^+^ subpopulation was examined by morphology observation and Cell Counting Kit-8 (CCK-8) assay on day 1, 3, and 5(Figure 3A-B). The results showed that both ADSCs and CD146^+^ cells adhered to the surface and proliferated well under standard culture conditions (Figure 3A). The latent phase and logarithmic phase lasted from day 1-3, and the stationary phase lasted from day 3-5. No significant difference was observed between ADSCs and CD146^+^ subpopulation during the entire period (Figure 3B). Cell viability (Figure 3E) and apoptosis (Figure 3C) were determined by flow cytometric analysis, and the percentages of cells in different stages were calculated (Figure 3D, F). The quantitative analysis suggested that the CD146^+^ subset maintained high cell viability (over 95%) and a low apoptotic rate after the enrichment confirming the good growth properties (Figure 3D, F).

### CD146^+^ subpopulation is distinct from hADSCs with a unique molecular signature

**Figure 4:**
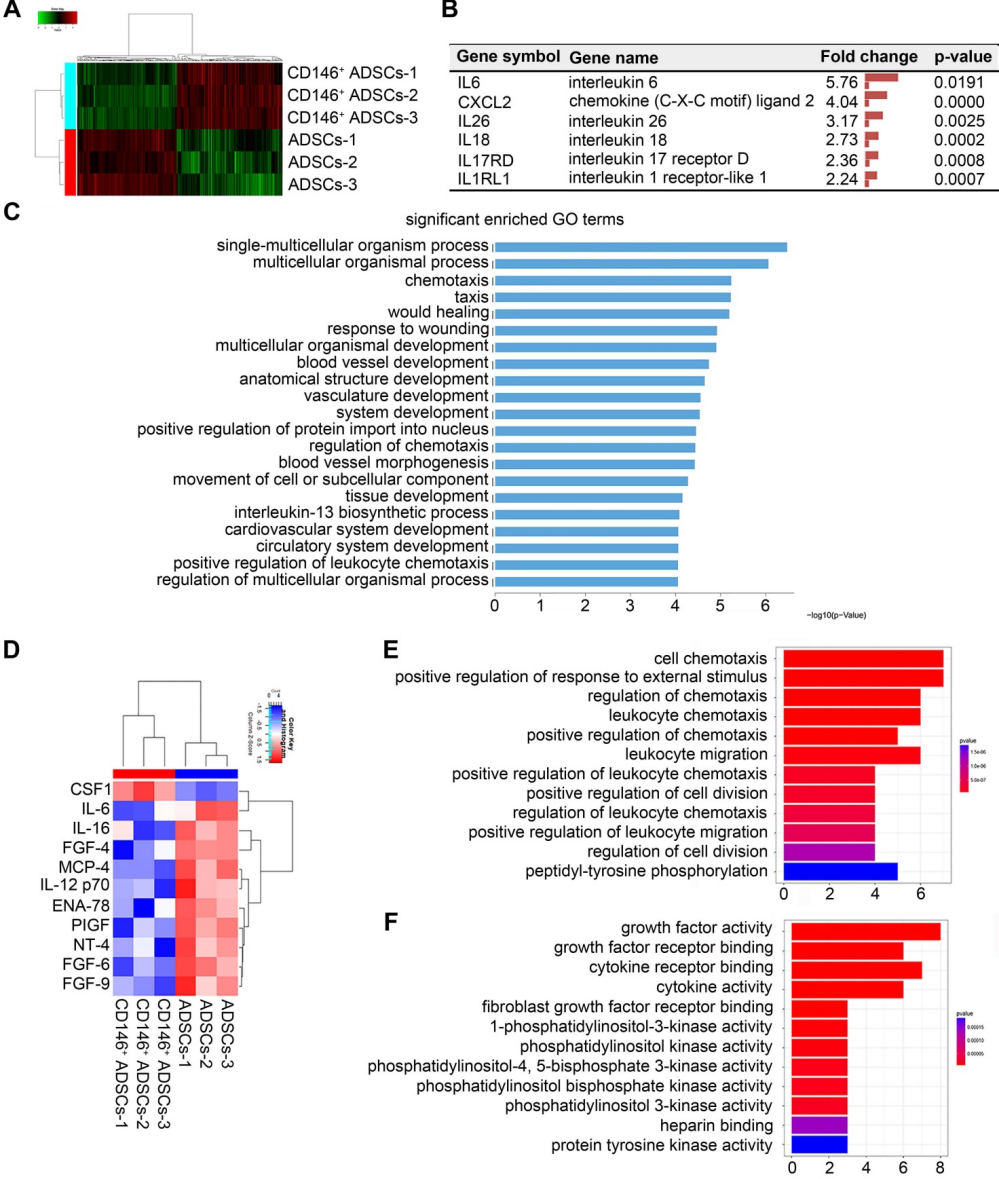
** Gene and protein expression of CD146^+^ ADSCs before and after MACS enrichment.** (A) Heatmap showing transcription expression profiles of ADSCs and the CD146^+^ subpopulation. Differentially expressed genes are shown. Each block represents the relative transcript level of an individual gene. Each column represents the profile of an individual group. (B) List of inflammation related genes that were significantly changed in CD146^+^ cells. (C) GO classification of genes by biological process. (D) Cytokine expression of ADSCs and CD146^+^ cells. Samples were collected from cell protein. Heatmap showing cytokine expression profiles of ADSCs and CD146^+^ cells. (E) GO classification of proteins by molecular function. (F) KEGG pathway of proteins.

To identify the molecular signature of the CD146^+^ subpopulation, we performed an mRNA and protein expression microarray analyses and compared the expression profiles of cells before and after sorting. In mRNA microarrays, a total of 428 differentially expressed genes were identified, of which 221 genes were up-regulated in CD146^+^ cells, and 208 were downregulated (Figure 4A). Differential gene expression was subjected to cluster analysis in which biological replicates in each group were clustered together (Figure 4A). Differentially expressed genes included immune and inflammatory genes such as IL6, CXCL2, IL1RL1, IL17RD, IL18, and IL26 (Figure 4B). Gene ontology (GO) classification of genes by biological processes showed differentially expressed genes were involved in multicellular organismal processes, such as chemotaxis, wound healing, blood vessels, and inflammatory processes (Figure 4C). Protein expression of ADSCs and CD146^+^ subpopulation was tested by using antibody microarrays. Among 80 chemokines and growth factors, 11 differentially expressed proteins were identified (Figure 4D). Differentially expressed chemokines included immune and inflammatory proteins such as IL6, IL12 and IL16. GO classification showed that differentially expressed proteins play a role in chemotaxis and cell division (Figure 4E-F). The combined gene and protein expression results indicated that, compared to hADSCs, the CD146^+^ subpopulation was a distinct population and had a molecular signature in inflammation functions before sorting.

### CD146+ subpopulation reduces inflammation of the articular cartilage during the early stages of cells injection

**Figure 5:**
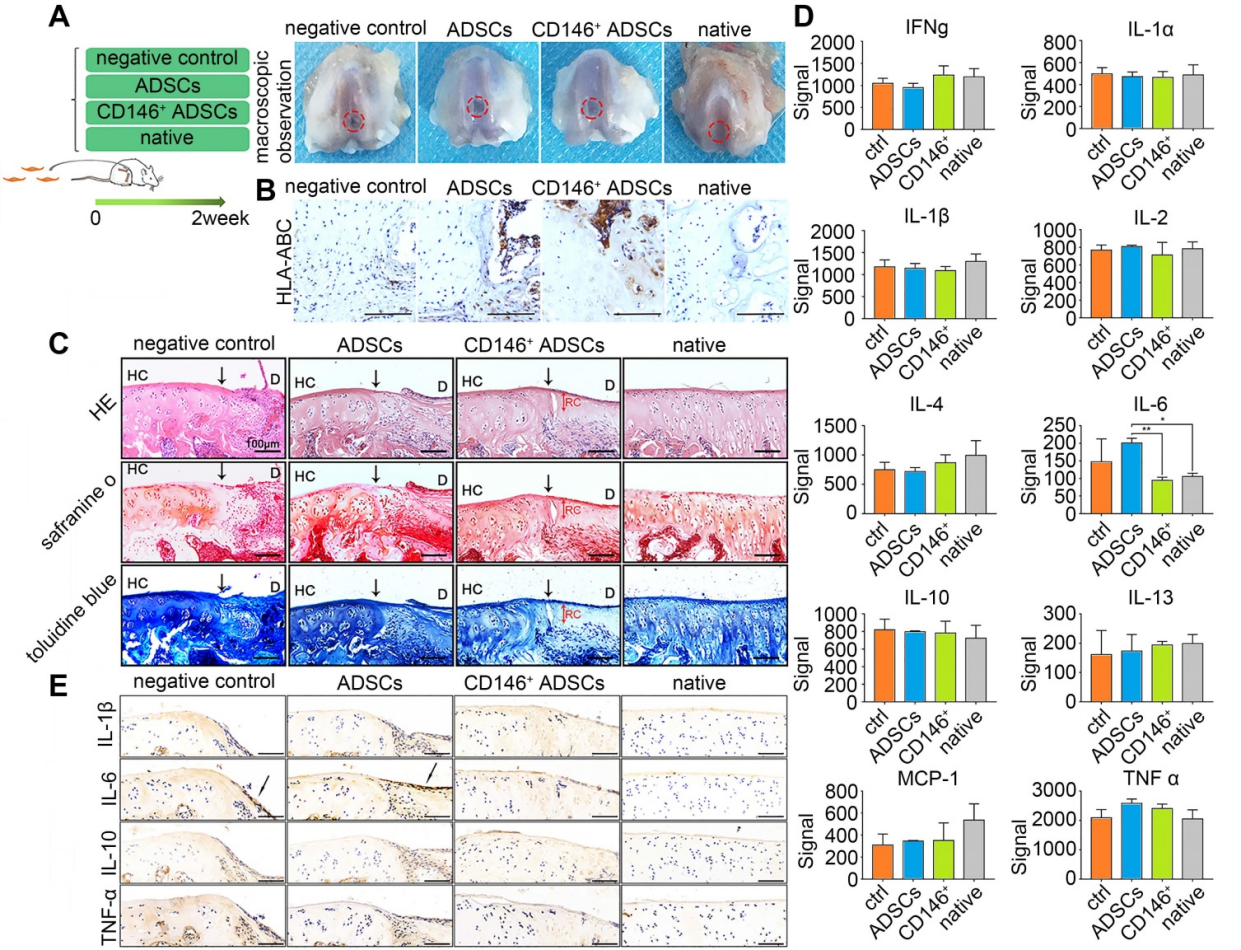
** Role of CD146^+^ ADSCs in cartilage microenvironment after joint cavity injection.** (A) Experimental design for cell transplantation and macroscopic analyses of femoral condyle cartilage. Rats were randomly allocated to four groups: defects treated with PBS (negative control), defects treated with ADSCs, defects treated with CD146^+^ cells, and the sham-operated group. At 2 weeks, rats were euthanized, and the distal femora were harvested. (B) Immunohistochemical staining of HLA-ABC. Scale bar: 100 μm. (C) Histological analysis of the defected area by H&E, Safranin O, and Sirius red staining. Black solid arrows denote the repair interface. Red solid arrows denote the depth of the repaired cartilage. HC, host cartilage; D, defect area; RC, repaired cartilage. Scale bar: 100 μm. (D) Analysis of inflammation related cytokine expressions. Results represent the mean ± standard error of the mean; the sample number was three. *P < 0.05, **P < 0.01. (E) Immunohistochemical staining of IL-1β, IL-6, IL-10 and TNF-α. Black solid arrows denote the positive expression of IL-6 in the repair interface Scale bar: 100 μm.

To elucidate the cellular functions *in vivo*, we employed a rat osteochondral defect model obtained by intra-articular injections of cells, and formulated four groups: defects with intra-articular injections of PBS (negative control), ADSCs, and CD146^+^ subpopulation, and the sham-operated group (Figure 5A). Macroscopic observation showed that the defect area could not be totally repaired in two weeks after injection in all the groups (Figure 5A). Immunohistochemical staining of HLA-ABC specially marked the location of the human cells in the early stages of cell implantation (Figure 5B). Results showed that both hADSCs and CD146^+^ subpopulation were recruited into the defect area. Histologically, although a time span of 2 weeks was too short for the repair, intra-articular injections of CD146^+^ subpopulation led to better improvements of the quality of osteochondral repair, with less inflammatory cells in the boundary between healthy tissue and the defected area (Figure 5C).

Inflammatory cytokines in articular cartilage and subchondral bone were further investigated. The expression level of IL-6 was significantly higher in the hADSCs group, than in the CD146^+^ group which was more similar to that in normal cartilage (Figure 5D). Also, the expression of cytokines such as IL-6, IL-1β, TNF-α, and IL-10 in the defect area was evaluated histologically. Two weeks after the injection, there was no significant difference in the expression of IL-1β, TNF-α and IL-10, while the expression of IL-6 was still present on the surface of the defect area in the negative control and ADSCs injected groups. Less expression of IL-6 was observed in the defect area of CD146^+^ cells injected groups (Figure 5E). Thus, the CD146^+^ subpopulation could reduce inflammation during the early stages of cells injection to promote cartilage repair.

### CD146^+^ cell-scaffold composites resulted in less subcutaneous inflammation

**Figure 6:**
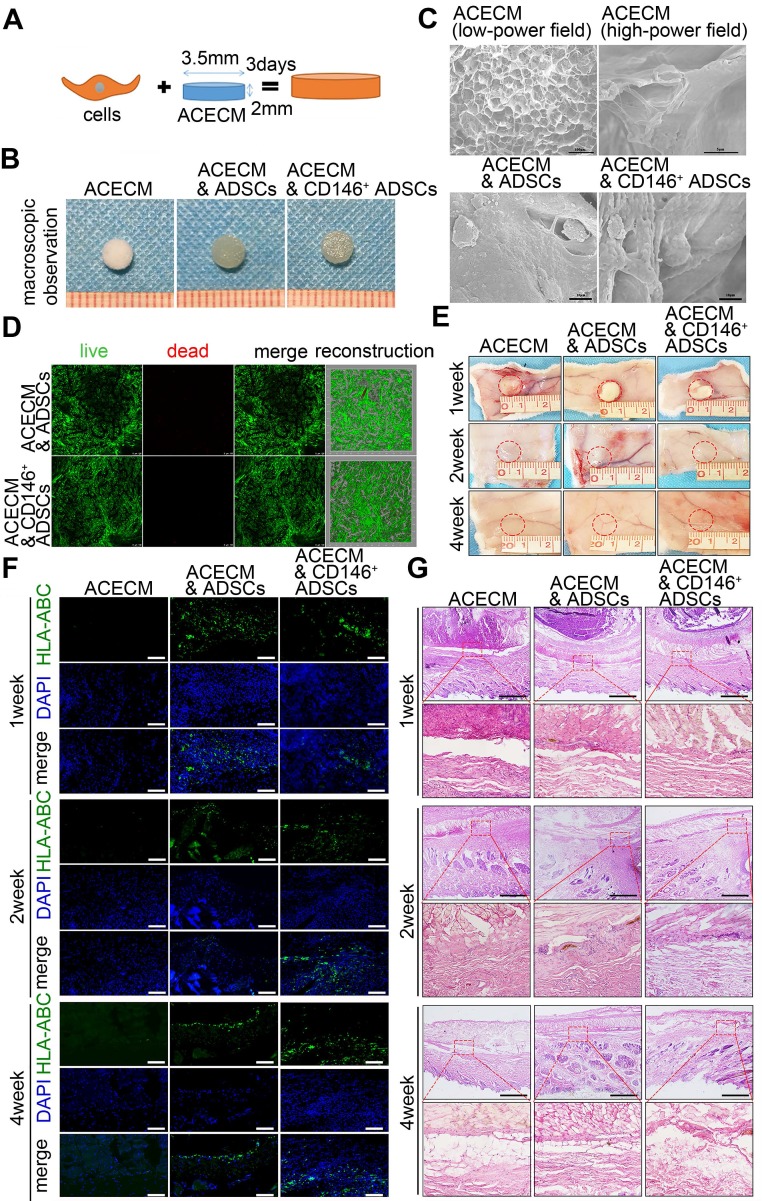
** Fabrication and biocompatibility of cell-scaffold composites.** (A) Experimental design of the cell-scaffold composites. ADSCs and CD146^+^ cells were seeded into an ACECM scaffold for 3 days. (B) Macroscopic features of the ACECM scaffold and two cell-scaffold composites. (C) SEM images of the ACECM scaffold and two cell-scaffold composites. (D) Live/dead cell analysis for the two cell-scaffold composites. Representative images show dead cells (red), live cells (green), and reconstruction images of the two cell-scaffold composites. (E) Photographs of the ACECM scaffold and two cell-scaffold composites subcutaneously implanted in the back of rabbits after 1 week, 2 weeks, and 4 weeks (the circle indicates the tissue surrounding the scaffold). (F) Immunofluorescent staining of HLA-ABC of the tissues surrounding the implant sites after 1 week, 2 weeks, and 4 weeks. Scale bar: 50 μm. (G) H&E staining of the tissues surrounding the implant sites after 1 week, 2 weeks, and 4 weeks. Scale bar: 100 μm.

Given that CD146^+^ subpopulation was associated with inflammation moderation of the cartilage microenvironment during the early stages of implantation, we developed ACECM scaffold composites for long-term cartilage repair. Same numbers of ADSCs and CD146^+^ subpopulation were seeded into ACECM scaffolds and cultured for 3 days *in vitro* (Figure 6A). Macroscopic photographs of the three groups are displayed Figure 6B. Scanning electron microscopy (SEM) images show the porous structure of the ACECM (Figure 6C). The inner walls of the evenly distributed pores increase the surface area and are suitable for cell adhesion. Both ADSCs and CD146^+^ subpopulation were located in the inner wall of the pores, maintained their phenotype, and performed normal function after 3 days of culturing (Figure 6C). Furthermore, cell viability was measured by live/dead cell staining (Figure 6D). Both CD146^+^ subpopulation and ADSCs proliferated and grew well in the three-dimensional porous scaffold and most viable cells exhibited a uniform distribution (Figure 6D). These results suggested that the ACECM is not toxic to cells and is suitable for cell growth as a useful biocarrier material for cartilage repair.

To further explore the *in vivo* degradation and biocompatibility of the composites, three types of cell-scaffold composites (ACECM scaffold, ADSCs & ACECM scaffold composites, and CD146^+^ subpopulation & ACECM scaffold composites) were subcutaneously implanted into the back skin of rabbits, and their role in modulating subcutaneous inflammation was investigated (Figure 6E-G). Macroscopic photographs showed that in 1, 2, and 4 weeks after implantation, cell-scaffold composites adhered to a layer of connective tissue and were degraded gradually *in vivo* (Figure 6E). Survival of human cells overtime was assessed by immunofluorescent staining of HLA-ABC. The results showed that ADSCs and CD146^+^ subpopulation retained high survival in 1, 2, and 4 weeks after implantation (Figure 6F). As per H&E staining, the appearance of inflammatory cells in the first week suggested an acute inflammatory response in all three groups (Figure 6G). From 2 to 4 weeks, the number of inflammatory cells in all three groups decreased gradually, reflecting the reduction in inflammatory response (Figure 6G). During the entire period of observation, the lowest number of inflammatory cells was observed in the CD146^+^ subpopulation & ACECM scaffold composite group, confirming that this scaffold composite caused the least subcutaneous inflammation.

### CD146^+^ subpopulation in combination with the ACECM scaffold improves cartilage regeneration

**Figure 7:**
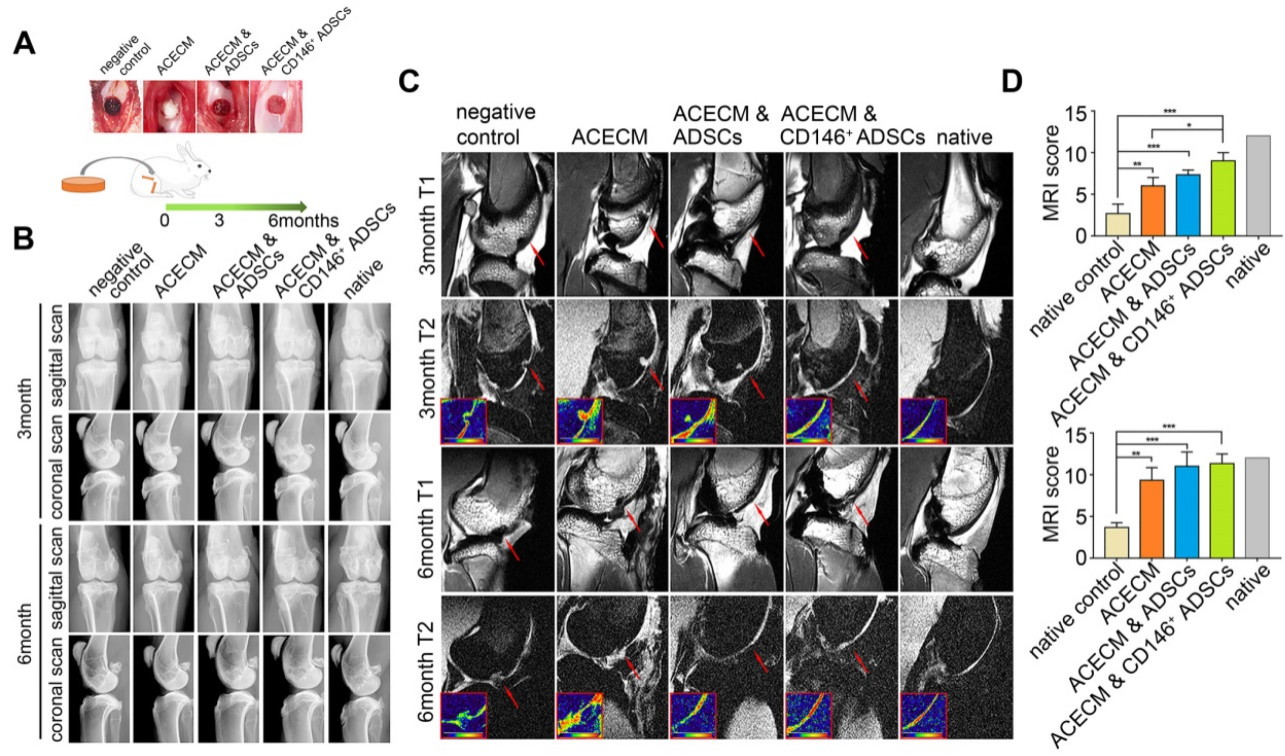
** Radiography assessment of the cartilage in rabbits with cell-scaffold composites treatment.** (A) Experimental design for the cartilage repair. The rabbits were randomly allocated into five groups: defects without treatment (negative control), defects treated with ACECM scaffold, defects treated with ACECMs and ADSCs, defects treated with ACECMs and CD146^+^ cells, and native group. (B) X-ray images of rabbit knees 3 and 6 months after surgery. (C) MRI of rabbit knees 3 months and 6 months after surgery. Red arrow indicates the defected area. The inset in the bottom left corner of each T2 mapping image shows the cartilage labeled by pseudo-color. (D) MRI scoring system for evaluation of cartilage repair after 3 and 6 months. Data are expressed as mean ± SD, *P < 0.05, **P < 0.01, ***P < 0.0001.

To verify the role of cell-scaffold composites in long term cartilage repair, we implanted them into the trochlear groove cartilage defect areas in rabbits. X-ray images revealed that there was no osteophyte formation and heterotopic ossification in any of the five groups during the experimental period (Figure 7B, Figure S3). At 3 months, new cartilage tissue was observed in the ACECM scaffold, ADSCs & ACECM scaffold composite, and CD146^+^ subpopulation & ACECM scaffold composite implanted groups by high-resolution MRI of the fresh whole knee joints, but immature cartilage signal was also present (Figure 7C). At 6 months, MRI images of the ADSCs & ACECM scaffold composite and CD146^+^ subpopulation & ACECM scaffold composite implanted groups were almost entirely filled with new cartilage tissue better than the ACECM scaffold implanted group (Figure 7C). During this period, no new tissue was found in the negative control group (Figure 7C). MRI score analysis also confirmed these results. As shown in Figure 7D, the score in CD146^+^ subpopulation & ACECM scaffold composite implanted groups was higher than the other groups at 3months. At 6 months, both ADSCs & ACECM scaffold composite and CD146^+^ subpopulation & ACECM scaffold composites implanted groups achieved a high score that was comparable to the native groups (Figure 7D).

**Figure 8:**
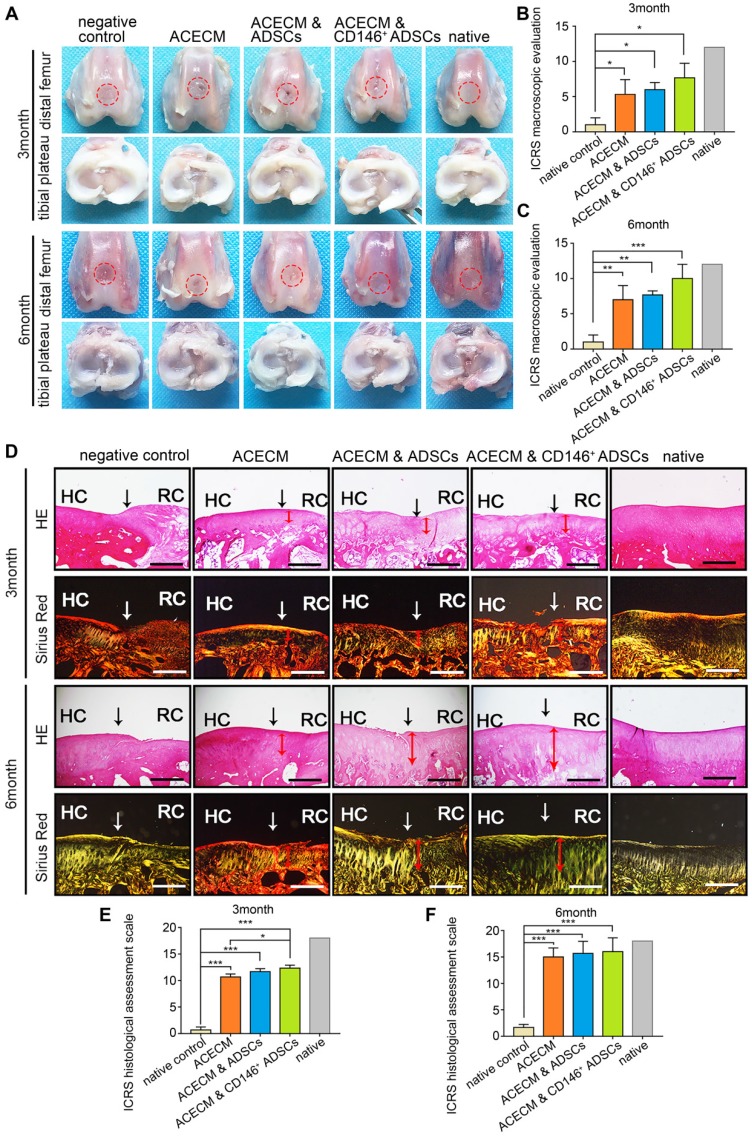
** Macroscopic and histological evaluation of cartilage repair after cell-scaffold composite treatment.** (A) Macroscopic observations of rabbit knees (distal femur and tibial plateau) 3 and 6 months after surgery. (B) (C) ICRS scoring system for macroscopic evaluation of cartilage repair after 3 months (B) and 6 months (C). Data are expressed as mean ± SD, *P < 0.05, **P < 0.01, ***P < 0.0001. (D) Histological analysis of the cartilage defect after 3 and 6 months by H&E and Sirius Red staining. Black solid arrows denote the repair interface. Red solid arrows denote the depth of the repaired cartilage. HC, host cartilage; RC, repaired cartilage. Scale bar: 200 μm. (E) (F) ICRS scoring system for histological evaluation of cartilage repair after 3 months (E) and 6 months (F).

The entire knee joint was dissected for macroscopic evaluation. No apparent inflammation and synovial hyperplasia were observed in any of the groups during the 6 months (Figure 8A). New cartilage generally formed from the edge of the defects in the ACECM scaffold and two cell-scaffold composite implanted groups from 3 to 6 months (Figure 8A). Generally, the shape of the new cartilage was better at 6 months compared with 3 months (Figure 8A). The regenerated cartilage in the CD146^+^ subpopulation & ACECM scaffold composite implanted group was almost similar to native cartilage after 6 months (Figure 8A). In the negative control group, the defect did not regrow during this period (Figure 8A). Consistent with the macroscopic results, the International Cartilage Repair Society (ICRS) macroscopic evaluation score for regenerated cartilage in the CD146^+^ subpopulation & ACECM scaffold composite implanted group was better than that of the other groups at 3 and 6 months (Figure 8B-C, Figure S4).

The thickness and structure of new cartilage in the defected area were visualized histologically (Figure 8D-F, Figure S5-8). The chondrocyte-like cells were visualized in the defected area in the ACECM, ADSCs & ACECM, and CD146^+^ subpopulation & ACECM scaffold composite implanted groups. Safranine O and Toluidine Blue stainings were negative in the defected area and positive in the regenerated hyaline cartilage. Generally, as the repair period progressed, the defect was gradually filled with new cartilage (Figure 8D-F, Figure S5-8). The new cartilage layer was thin in the ACECM scaffold implanted group compared with the ADSCs & ACECM, and CD146^+^ subpopulation & ACECM scaffold composite implanted groups. However, no new cartilage layer was found in the negative control group at both 3 and 6 months (Figure 8D-F, Figure S5-8). At 6 months, the thickness of the new cartilage layer in the CD146^+^ subpopulation & ACECM scaffold composite implanted group was similar to that of the native cartilage, with less void space and a regular surface. Sirius Red staining also highlighted a tangled arrangement of collagen fibers in the CD146^+^ subpopulation & ACECM scaffold composite implanted group at 6 months (Figure. 8D-F, Figure S6-9). Better regeneration of the cartilage in the CD146^+^ subpopulation & ACECM scaffold composites implanted group was also confirmed by the International Cartilage Repair Society (ICRS) macroscopic evaluation score.

## Discussion

It has long been known that MSCs comprise a heterogeneous population, which impedes their application in regenerative medicine 22. Studies found that some subpopulations have enhanced capacity for cartilage formation. For example, Dickinson et al. reported the subpopulation with the receptor tyrosine kinase-like orphan receptor 2 (ROR2) as a cell surface marker is effective at making cartilage. The application of subpopulations is emerging as a novel idea for cartilage repair 41.

As a surface marker expressed by pericytes and the smooth muscle cells of blood vessels, CD146 plays a critical role in cell adhesion, embryonic development, immune response, angiogenesis, and cancer 42-45. Previous studies have shown that CD146 defines a functional subset of progenitor cell populations and exhibit higher developmental potentials 27, 46-47. *In vivo*, CD146^+^ cells surround endothelial cells populating the vascular intima. With specific adhesion and migration properties, CD146^+^ cells can regulate blood vessel stability/integrity as well as the proliferation and motility of adjacent endothelial cells 48. Both *in vitro* and *in vivo* studies provided evidence that CD146+ perivascular cells, as constituents of MSCs, and play a critical role during wound healing 49. For example, increased number of CD146^+^ cells by activation of PDGF-BB/ PDGFRβ signaling pathway has been reported during wound healing *in vivo* 50. CD146^+^ cells have also been shown to promote wound healing by secreting high levels of cytokines such as KGF, vascular endothelial growth factor (VEGF), heparin binding-epidermal growth factor (HB-EGF) and basic-fibroblast growth factor (bFGF) 51. CD146+ cells isolated from human lipoaspirate showed immunomodulatory effect during bone formation 52. In another study, CD146^+^ cells have been shown to induce increased cord formation 53. In a degenerated intervertebral disc model, CD146^+^ MSCs showed greater migration potential for re-population 54. However, the role of CD146^+^ cells as a seed cell type in cartilage tissue engineering is unclear.

In this study, a CD146^+^ subpopulation of hADSCs was selected, and applied for cartilage repair as evidence from both *in vitro* and *in vivo* experiments supports its role in cartilage repair. The expressions of MSC related surface markers were almost unchanged after the enrichment. Pericytes as progenitor cells ubiquitously express surface markers such as NG2, PDGFR-β, and RGS5 in almost all organs 27, 55. Consistent with previous reports, the CD146^+^ population from hADSCs expressed high levels of other pericyte markers, was distinct from hADSCs, and maintained their progenitor cell character. Immunogenicity affects the outcome of cell transplantation. MSCs can avoid allogeneic rejection, prolong graft survival, and induce tolerance in humans and in animal models. When CD146^+^ cells were sorted from human adipose tissue for bone formation in mice, no immunological rejection was observed 52. MHC class II proteins are potent alloantigens. Under non-inflammatory conditions, human MSCs are MHC-II negative, supporting a role for MSC in having reduced immunogenicity through the control of alloantigen expression 56-60. The expression of MHC-I in the CD146^+^ subpopulation is significantly lower than in hADSCs with no MHC-II expression, supporting a role for the CD146^+^ subpopulation with reduced immunogenicity through the control of alloantigen expression. Collectively, the CD146+ population has immune characteristics to avoid allogeneic rejection.

Previous studies found that CD146 was associated with proliferation and expressed at high levels in nearly all clonal MSCs, while only a minority of nonclonal MSCs expressed this marker 31. In this study, we observed high proliferation rate, cell viability, and low apoptosis verifying the cell survival capability of CD146^+^ cells, and supporting their suitability for transplantation. It was important to elucidate the characteristic features of CD146^+^ cells at the molecular level. Our microarray data highlighted a variety of functions of CD146^+^ cells in chemotaxis, wound healing, blood vessels, and inflammatory processes, confirming that the CD146^+^ population is functionally distinct from the hADSCs.

Cartilage defects typically lead to posttraumatic inflammation and represent a significant challenge in cartilage repair 61. It has been shown that MSCs can promote tissue repair by modulating inflammation 62. *In vitro*, both gene and protein results revealed a differential expression profile of inflammation related cytokines between CD146^+^ cells and hADSCs. To investigate the specific cellular functions in the cartilage microenvironment *in vivo*, we injected CD146^+^ cells into the joint cavity. In the defect area, CD146^+^ cells did not lead to higher expression levels of IL-6 compared with hADSCs. This result is consistent with a previous report showing that a CD146^+^ subpopulation from human umbilical cord cells provided an anti-inflammatory protective microenvironment by suppressing IL-6 34. Another study also reported that CD146^+^ cell implantation reduced Ly-6G^+^ and F4/80^+^ cell infiltration in bone 52. Thus, CD146^+^ population after enrichment plays a better role in modulating inflammation.

The clinical applications of MSCs is often limited by their poor viability at the site of injury caused by the unfavorable microenvironment and to anoikis driven by the loss of cell adhesion 63. To increase the viability of cells, we fabricated a cell ACECM scaffold composites to create a proper microenvironment for cell adhesion. For long-term cartilage regeneration, ACECM is the best biomaterial to mimic native cartilage and drive tissue homeostasis and regeneration because of the presence of bioactive molecules 64. Also, ACECM is biodegradable and does not elicit adverse immune responses 64. In this study, several findings supported good biocompatibility of cell ACECM scaffold composites. First, SEM images showed the loose and porous structure of ACECM that is suitable for cell adhesion. ADSCs and CD146^+^ cells were located on the inner wall of pores, maintained their phenotype, and functioned normally after culturing. Second, CD146^+^ cells and ADSCs proliferated and grew well in the three-dimensional porous scaffold, and the most viable cells exhibited a uniform distribution. Third, when the degradability of the cell-scaffold composites *in vivo* was tested by subcutaneous implantation in rabbits, they displayed good biocompatibility without any visible foreign body reaction. Finally, CD146^+^ subpopulation survived even 4 weeks after implantation confirming that ACECM scaffold created a proper microenvironment for cell adhesion. The lower number of inflammatory cells confirmed that the CD146^+^ cells caused a more significant reduction in subcutaneous inflammation than the more heterogeneous hADSCs. These results were consistent with the previous finding that CD146^+^ cells combined with collagen scaffold did not induce an early inflammatory infiltrate during bone formation 52.

In the rabbit implantation experiment, the outcome observed in the CD146^+^ subpopulation & ACECM scaffold composite implanted group was superior to all other groups as confirmed by imaging, macroscopic observation, and histological examination. The regenerative capacity of tissues depends on the degree of the inflammatory response 65. We hypothesized that CD146^+^ subpopulation would play a superior role in inhibiting inflammation and promoting long term outcome. ACECM can stimulate growth and proliferation, and ensure the normal functioning of CD146^+^ subpopulation. Enhanced proliferation of the CD146^+^ subpopulation can offset the dysfunction caused by the insufficient number of cells. Also, direct differentiation of MSCs into chondrocytes is one of the key factors for cartilage repair. In this context, the differentiation ability of the CD146^+^ subpopulation has been demonstrated in previous studies 27, 66-68. CD146^+^ cells can directly differentiate into chondrocytes and promote cartilage repair. However, CD146 is not a specific chondrogenesis marker, although some studies have shown that CD146^+^ subset has better chondrogenic differentiation ability 67. It is of note that CD146^-^ subset can also differentiate into chondrocytes 54. CD146^-^ cells are also heterogeneity and contain progenitors cells of MSC. For example, CD34+ cells, resides in the outmost layer of blood vessels, also natively express MSC markers and give rise in culture to progenitors cells of MSC 69. The regulation of chondrogenic differentiation in CD146^+^ and CD146^-^ cells remains to be further investigated.

The quantity and stability of cells are important factors that affect subpopulation-based therapy. CD146+ cells sorted from fetal and adult muscle, midterm placenta, fetal skin, fetal pancreas, bone marrow, and adipose tissue can retain the phenotype, exhibit osteogenic, chondrogenic, and adipogenic potentials and express MSC and pericyte markers 27. For example, muscle-derived CD146+ cells stably expressed pericyte markers such as NG2, CD146, and α-SMA after 4, 8, or 14 passages 27. CD146+ cells from mesenchymoangioblast can be cultured in the presence of platelet-derived growth factor (PDGF)-BB for up to 12 passages with gradual senescence observed during 8-12 passages 70. In our study, we used cells for *in vivo* cartilage repair without long term culture *in vitro*. Compared with bone marrow MSCs, large numbers of ADSCs can be obtained from lipoaspirate in one attempt with less pain. In the rat model, CD146^+^ cells were injected into the joint cavity immediately after MACS enrichment without *in vitro* culture. In the rabbit model, CD146^+^ cells were cultured 3 days before *in vivo* implantation. In future clinical applications, sufficient numbers of CD146^+^ subpopulation can be obtained without requiring serial sub-culturing *in vitro*. Thus, subpopulation-ased strategy in this study is more feasible for clinical translation. Another important issue that needs to be explored in the future is the variability of CD146^+^ cells between donors to enable selection of the proper subpopulation for therapy.

## Conclusions

Our study highlights the importance of a subpopulation-based MSCs therapy in regenerative medicine. Although it has long been known that MSCs are heterogeneous and subpopulations vary functionally, the precise roles of different subpopulations in cartilage tissue repair has remained elusive. Our ultimate goal is to optimize cartilage structure and function utilizing specific properties of MSC subtypes and make tissue engineering more effective. Thus, uncovering the functional characteristics of specific subtypes of MSCs is a new direction for cell-based tissue engineering research and can potentially further the development of precision medicine.

## Supplementary Material

Supplementary figures and tables.

## References

[1] Buckwalter JA, Mankin HJ (1998). Articular cartilage: degeneration and osteoarthritis, repair, regeneration, and transplantation. Instr Course Lect.

[2] O'Driscoll SW (1998). The healing and regeneration of articular cartilage. J Bone Joint Surg Am.

[3] Adkisson HD 4th, Martin JA, Amendola RL, Milliman C, Mauch KA, Katwal AB (2010). The potential of human allogeneic juvenile chondrocytes for restoration of articular cartilage. Am J Sports Med.

[4] Beiser IH, Kanat IO (1990). Subchondral bone drilling: a treatment for cartilage defects. J Foot Surg.

[5] Steadman JR, Briggs KK, Rodrigo JJ, Kocher MS, Gill TJ, Rodkey WG (2003). Outcomes of microfracture for traumatic chondral defects of the knee: average 11-year follow-up. Arthroscopy.

[6] Albright JC, Daoud AK (2017). Microfracture and Microfracture Plus. Clin Sports Med.

[7] Kraeutler MJ, Houck DA, Mcqueen MB, Mccarty EC, Schrock JB (2017). A Cost-Effectiveness Analysis of Surgical Treatment Modalities for Chondral Lesions of the Knee: Microfracture, Osteochondral Autograft Transplantation, and Autologous Chondrocyte Implantation.

[8] Steinert AF, Ghivizzani SC, Rethwilm A, Tuan RS, Evans CH, Nöth U (2007). Major biological obstacles for persistent cell-based regeneration of articular cartilage. Arthritis Res Ther.

[9] Wang M, Yuan Z, Ma N, Hao C, Guo W, Zou G (2017). Advances and Prospects in Stem Cells for Cartilage Regeneration. Stem Cells Int.

[10] Li X, Wang M, Jing X, Guo W, Hao C, Zhang Y (2018). Bone Marrow- and Adipose Tissue-Derived Mesenchymal Stem Cells: Characterization, Differentiation, and Applications in Cartilage Tissue Engineering. Crit Rev Eukaryot Gene Expr.

[11] Lee WY, Wang B (2017). Cartilage repair by mesenchymal stem cells: Clinical trial update and perspectives. J Orthop Translat.

[12] Kong L, Zheng LZ, Qin L, Ho KKW (2017). Role of mesenchymal stem cells in osteoarthritis treatment. J Orthop Translat.

[13] Fellows CR, Matta C, Zakany R, Khan IM, Mobasheri A (2016). Adipose, Bone Marrow and Synovial Joint-Derived Mesenchymal Stem Cells for Cartilage Repair. Front Genet.

[14] Shiraishi K, Kamei N, Takeuchi S, Yanada S, Mera H, Wakitani S (2017). Quality Evaluation of Human Bone Marrow Mesenchymal Stem Cells for Cartilage Repair. Stem Cells Int.

[15] Francis SL, Duchi S, Onofrillo C, Bella CD, Choong PFM (2018). Adipose-Derived Mesenchymal Stem Cells in the Use of Cartilage Tissue Engineering: The Need for a Rapid Isolation Procedure. Stem Cells Int.

[16] Morito T, Muneta T, Hara K, Ju YJ, Mochizuki T, Makino H (2008). Synovial fluid-derived mesenchymal stem cells increase after intra-articular ligament injury in humans. Rheumatology.

[17] Kuznetsov SA, Mankani MH, Gronthos S, Satomura K, Bianco P, Robey PG (2001). Circulating Skeletal Stem Cells. J Cell Biol.

[18] Zhang Y, Liu S, Guo W, Wang M, Hao C, Gao S (2018). Human Umbilical Cord Wharton's Jelly Mesenchymal Stem Cells Combined with an Acellular Cartilage Extracellular Matrix Scaffold Improve Cartilage Repair Compared with Microfracture in a Caprine Model. Osteoarthritis Cartilage.

[19] Ps ITA, Scherjon SA, Kleijburg-Van dKC, Noort WA, Claas FH, Willemze R (2003). Amniotic fluid as a novel source of mesenchymal stem cells for therapeutic transplantation. Blood.

[20] Nogami M, Tsuno H, Koike C, Okabe M, Yoshida T, Seki S (2012). Isolation and characterization of human amniotic mesenchymal stem cells and their chondrogenic differentiation. Transplantation.

[21] Pérezsilos V, Camachomorales A, Fuentesmera L (2016). Mesenchymal Stem Cells Subpopulations: Application for Orthopedic Regenerative Medicine. Stem Cells Int.

[22] Li Z, Zhang C, Weiner LP, Zhang Y, Zhong JF (2013). Molecular characterization of heterogeneous mesenchymal stem cells with single-cell transcriptomes. Biotechnol Adv.

[23] Ouhtit A, Gaur RL, Abd Elmageed ZY, Fernando A, Thouta R, Trappey AK (2009). Towards understanding the mode of action of the multifaceted cell adhesion receptor CD146. Biochim Biophys Acta.

[24] Crisan M, Corselli M, Chen WC, Péault B (2012). Perivascular cells for regenerative medicine. J Cell Mol Med.

[25] Lehmann JM, Riethmüller G, Johnson JP (1989). MUC18, a marker of tumor progression in human melanoma, shows sequence similarity to the neural cell adhesion molecules of the immunoglobulin superfamily. Proc Natl Acad Sci U S A.

[26] Chen J, Luo Y, Huang H, Wu S, Jing F, Zhang J (2017). CD146 is essential for PDGFRβ-induced pericyte recruitment.

[27] Crisan M, Yap S, Casteilla L, Chen CW, Corselli M, Park TS (2008). A perivascular origin for mesenchymal stem cells in multiple human organs. Cell Stem Cell.

[28] Zhao H, Feng J, Seidel K, Shi S, Klein O, Sharpe P (2014). Secretion of shh by a neurovascular bundle niche supports mesenchymal stem cell homeostasis in the adult mouse incisor. Cell Stem Cell.

[29] Horl S, Ejaz A, Ernst S, Mattesich M, Kaiser A, Jenewein B (2017). CD146 (MCAM) in human cs-DLK1(-)/cs-CD34(+) adipose stromal/progenitor cells. Stem Cell Res.

[30] Spitzer TLB, Rojas A, Zelenko Z, Aghajanova L, Erikson DW, Barragan F (2011). Perivascular human endometrial mesenchymal stem cells express pathways relevant to self-renewal, lineage specification, and functional phenotype. Biol Reprod.

[31] Sacchetti B, Funari A, Michienzi S, Di CS, Piersanti S, Saggio I (2007). Self-renewing osteoprogenitors in bone marrow sinusoids can organize a hematopoietic microenvironment. Cell.

[32] Luo Y, Zheng C, Zhang J, Lu D, Zhuang J, Xing S (2012). Recognition of CD146 as an ERM-binding protein offers novel mechanisms for melanoma cell migration. Oncogene.

[33] Hagmann S, Frank S, Gotterbarm T, Dreher T, Eckstein V, Moradi B (2014). Fluorescence activated enrichment of CD146+ cells during expansion of human bone-marrow derived mesenchymal stromal cells augments proliferation and GAG/DNA content in chondrogenic media. BMC Musculoskelet Disord.

[34] Wu CC, Liu FL, Sytwu HK, Tsai CY, Chang DM (2016). CD146 + mesenchymal stem cells display greater therapeutic potential than CD146 - cells for treating collagen-induced arthritis in mice. Stem Cell Res Ther.

[35] Su X, Zuo W, Wu Z, Chen J, Wu N, Ma P (2015). CD146 as a new marker for an increased chondroprogenitor cell sub-population in the later stages of osteoarthritis. J Orthop Res.

[36] Frese L, Dijkman PE, Hoerstrup SP (2016). Adipose Tissue-Derived Stem Cells in Regenerative Medicine. Transfus Med Hemother.

[37] Zhou W, Lin J, Zhao K, Jin K, He Q, Hu Y (2019). Single-Cell Profiles and Clinically Useful Properties of Human Mesenchymal Stem Cells of Adipose and Bone Marrow Origin. Am J Sports Med.

[38] Eisen MB, Spellman PT, Brown PO, Botstein D (1998). Cluster analysis and display of genome-wide expression patterns. Proc Natl Acad Sci U S A.

[39] Yang Q, Peng J, Guo Q, Huang J, Zhang L, Yao J (2008). A cartilage ECM-derived 3-D porous acellular matrix scaffold for in vivo cartilage tissue engineering with PKH26-labeled chondrogenic bone marrow-derived mesenchymal stem cells. Biomaterials.

[40] Saw KY, Anz A, Siew-Yoke Jee C, Merican S, Ching-Soong Ng R, Roohi SA (2013). Articular cartilage regeneration with autologous peripheral blood stem cells versus hyaluronic acid: a randomized controlled trial. Arthroscopy.

[41] Dickinson SC, Sutton CA, Brady K, Salerno A, Katopodi T, Williams RL (2017). The Wnt5a Receptor, Receptor Tyrosine Kinase-Like Orphan Receptor 2, Is a Predictive Cell Surface Marker of Human Mesenchymal Stem Cells with an Enhanced Capacity for Chondrogenic Differentiation. Stem cells.

[42] Wang Z, Yan X (2013). CD146, a multi-functional molecule beyond adhesion. Cancer Lett.

[43] Ye Z, Zhang C, Tu T, Sun M, Liu D, Lu D (2013). Wnt5a uses CD146 as a receptor to regulate cell motility and convergent extension. Nat Commun.

[44] Kang Y, Wang F, Feng J, Yang D, Yang X, Yan X (2006). Knockdown of CD146 reduces the migration and proliferation of human endothelial cells. Cell Res.

[45] Tao T, Zhang C, Yan H, Luo Y, Kong R, Wen P (2015). CD146 acts as a novel receptor for netrin-1 in promoting angiogenesis and vascular development. Cell Res.

[46] Harkness L, Zaher W, Ditzel N, Isa A, Kassem M (2016). CD146/MCAM defines functionality of human bone marrow stromal stem cell populations. Stem Cell Res Ther.

[47] Tsang WP, Shu Y, Kwok PL, Zhang F, Lee KK, Tang MK (2013). CD146+ human umbilical cord perivascular cells maintain stemness under hypoxia and as a cell source for skeletal regeneration. PLoS One.

[48] Armulik A, Abramsson A, Betsholtz C (2005). Endothelial/pericyte interactions. Circ Res.

[49] Chen WCW, Park TS, Murray IR, Zimmerlin L, Lazzari L, Huard J (2013). Cellular kinetics of perivascular MSC precursors. Stem Cells Int.

[50] Lindahl P, Johansson BR, Leveen P, Betsholtz C (1997). Pericyte loss and microaneurysm formation in PDGF-B-deficient mice. Science.

[51] Chen CW, Montelatici E, Crisan M, Corselli M, Huard J, Lazzari L (2009). Perivascular multi-lineage progenitor cells in human organs: regenerative units, cytokine sources or both?. Cytokine Growth Factor Rev.

[52] Meyers CA, Xu J, Zhang L, Asatrian G, Ding C, Yan N (2018). Early Immunomodulatory Effects of Implanted Human Perivascular Stromal Cells During Bone Formation. Tissue Eng Part A.

[53] Wang Y, Xu J, Chang L, Meyers CA, Zhang L, Broderick K (2019). Relative contributions of adipose-resident CD146(+) pericytes and CD34(+) adventitial progenitor cells in bone tissue engineering. NPJ Regen Med.

[54] Wangler S, Menzel U, Li Z, Ma J, Hoppe S, Benneker LM (2019). CD146/MCAM distinguishes stem cell subpopulations with distinct migration and regenerative potential in degenerative intervertebral discs.

[55] James AW, Hindle P, Murray IR, West CC, Tawonsawatruk T, Shen J (2016). Pericytes for the treatment of orthopedic conditions.

[56] Zhang S, Jiang YZ, Zhang W, Chen L, Tong T, Liu W (2013). Neonatal Desensitization Supports Long-Term Survival and Functional Integration of Human Embryonic Stem Cell-Derived Mesenchymal Stem Cells in Rat Joint Cartilage Without Immunosuppression. Stem Cells Dev.

[57] Tse WT, Pendleton JD, Beyer WM, Egalka MC, Guinan EC (2003). Suppression of allogeneic T-cell proliferation by human marrow stromal cells: implications in transplantation. Transplantation.

[58] Majumdar MK, Keane-Moore M, Buyaner D, Hardy WB, Moorman MA, Mcintosh KR (2003). Characterization and functionality of cell surface molecules on human mesenchymal stem cells. J Cell Mol Med.

[59] Niemeyer P, Kornacker M, Mehlhorn A, Seckinger A, Vohrer J, Schmal H (2007). Comparison of immunological properties of bone marrow stromal cells and adipose tissue-derived stem cells before and after osteogenic differentiation in vitro. Tissue Eng.

[60] Liu S, Yuan M, Hou K, Li Z, Zheng X, Zhao B (2012). Immune characterization of mesenchymal stem cells in human umbilical cord Wharton's jelly and derived cartilage cells. Cell Immunol.

[61] Zhang Y, Pizzute T, Pei M (2014). Anti-inflammatory strategies in cartilage repair. Tissue Eng Part B Rev.

[62] Glenn JD, Whartenby KA (2014). Mesenchymal stem cells: Emerging mechanisms of immunomodulation and therapy. World J Stem Cells.

[63] Lee S, Choi E, Cha MJ, Hwang KC (2015). Cell adhesion and long-term survival of transplanted mesenchymal stem cells: a prerequisite for cell therapy. Oxid Med Cell Longev.

[64] Benders KE, van Weeren PR, Badylak SF, Saris DB, Dhert WJ, Malda J (2013). Extracellular matrix scaffolds for cartilage and bone regeneration. Trends Biotechnol.

[65] Eming SA, Wynn TA, Martin P (2017). Inflammation and metabolism in tissue repair and regeneration. Science.

[66] Tawonsawatruk T, West CC, Murray IR, Soo C, Peault B, Simpson AH (2016). Adipose derived pericytes rescue fractures from a failure of healing-non-union. Sci Rep.

[67] Wu CC, Liu FL, Sytwu HK, Tsai CY, Chang DM (2016). CD146+ mesenchymal stem cells display greater therapeutic potential than CD146- cells for treating collagen-induced arthritis in mice. Stem Cell Res Ther.

[68] Alvarez R, Lee H-L, Wang C-Y, Hong C (2015). Characterization of the osteogenic potential of mesenchymal stem cells from human periodontal ligament based on cell surface markers. Int J Oral Sci.

[69] Corselli M, Chen C-W, Sun B, Yap S, Rubin JP, Péault B (2012). The tunica adventitia of human arteries and veins as a source of mesenchymal stem cells. Stem Cells Dev.

[70] Kumar A, D'Souza SS, Moskvin OV, Toh H, Wang B, Zhang J (2017). Specification and Diversification of Pericytes and Smooth Muscle Cells from Mesenchymoangioblasts. Cell Rep.

