# Supplementary material for: Enrichment of CD146+ Adipose-Derived Stem Cells in Combination with Articular Cartilage Extracellular Matrix Scaffold Promotes Cartilage Regeneration
Source: Theranostics. 2019 Jul 9;9(17):5105–21. doi: 10.7150/thno.33904 (PMC6691381; doi:10.7150/thno.33904)
Supplement: Supplementary file 1 — Supplementary figures and tables. [file thnov09p5105s1.pdf]

Enrichment of CD146$^+$ adipose-derived stem cells in combination with articular cartilage extracellular matrix scaffold promotes cartilage regeneration

Supplementary materials

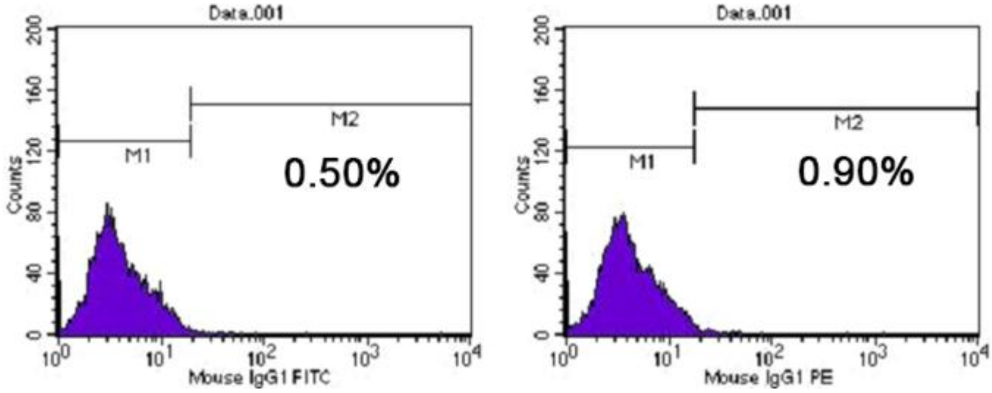

Figure S1. Isotype control of flow cytometry

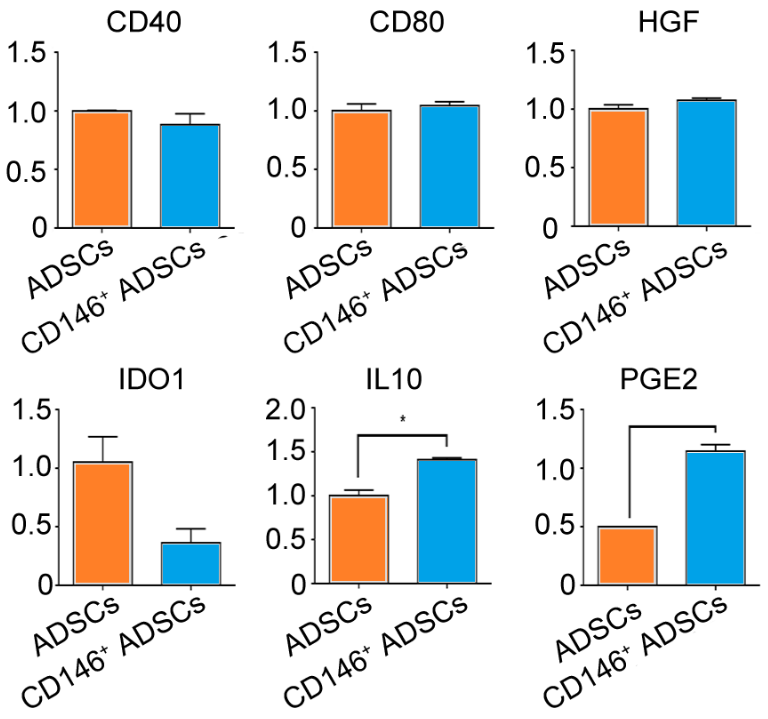

Figure S2. Quantification of mRNA levels of *CD40*, *CD80*, *HGF*, *IDO1*, *IL10*, and *PGE2* in CD146$^+$ cells and hADSCs by qRT-PCR. Data represent mean ± SD from three independent experiments.

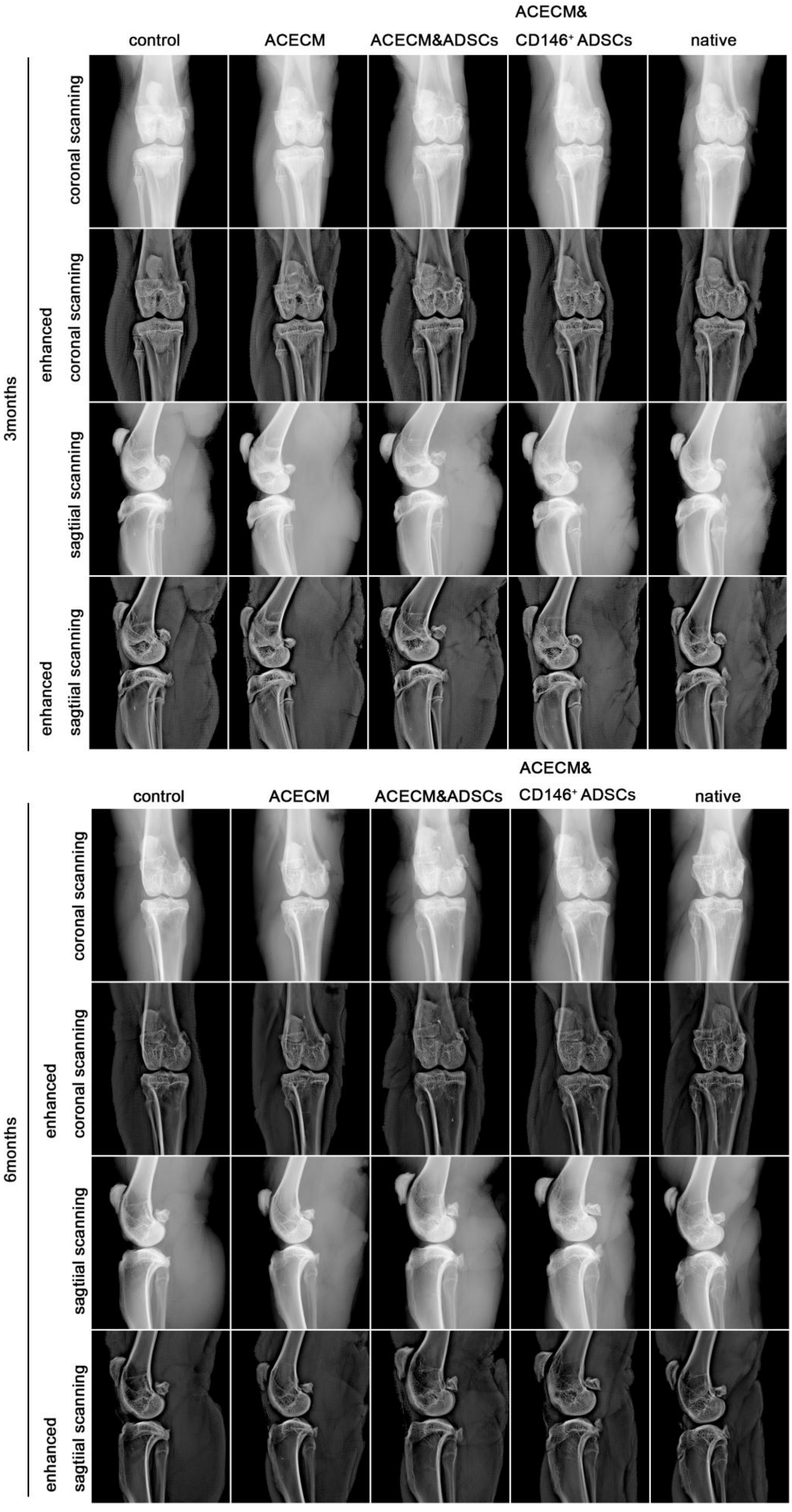

Figure S3. X-ray images of rabbit knees at 3 months and 6 months after surgery.

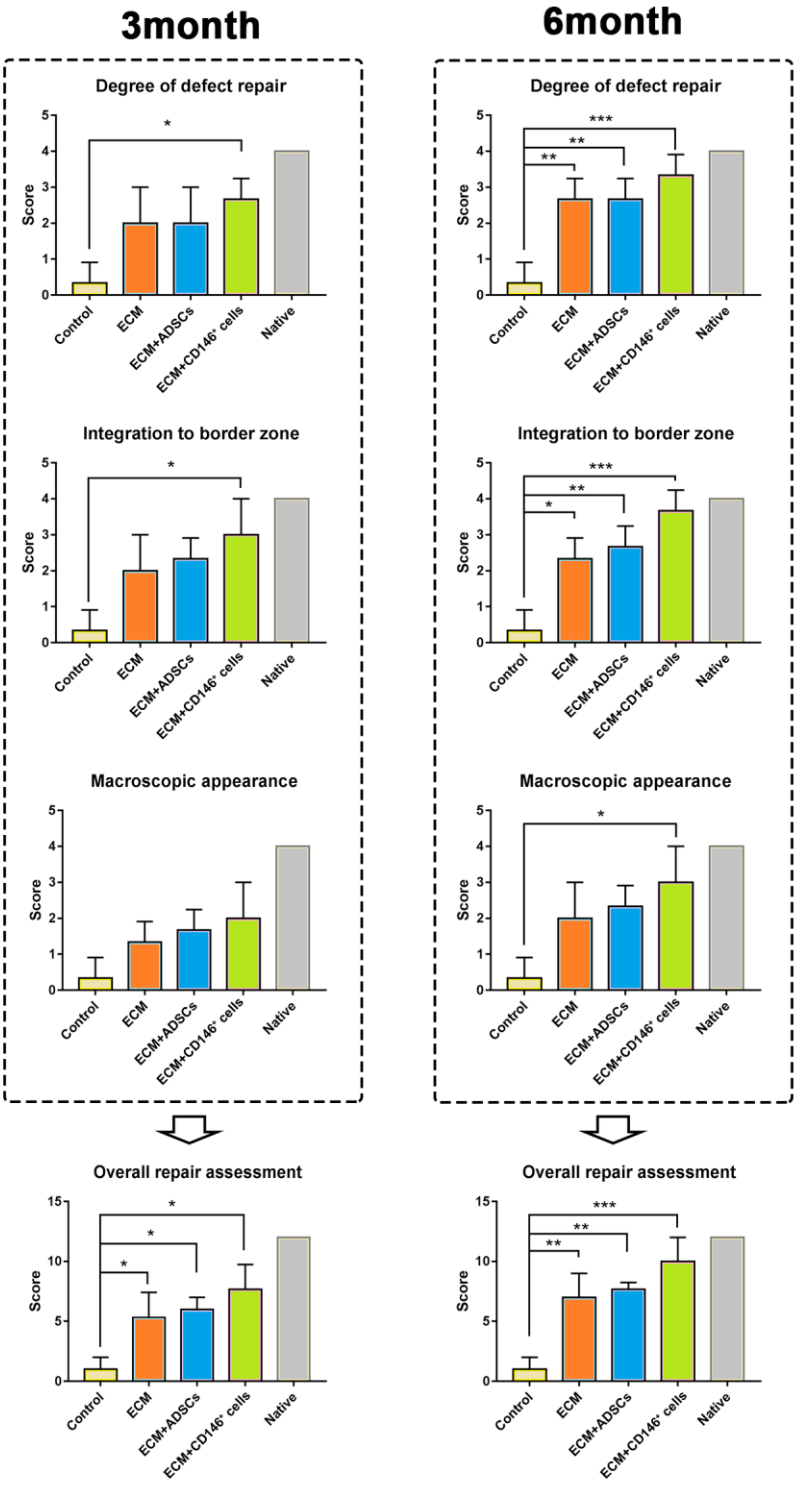

Figure S4. ICRS scoring system for macroscopic evaluation of cartilage repair after 3 and 6 months. The results represent the mean ± standard error of the mean. *P < 0.05, **P < 0.01.

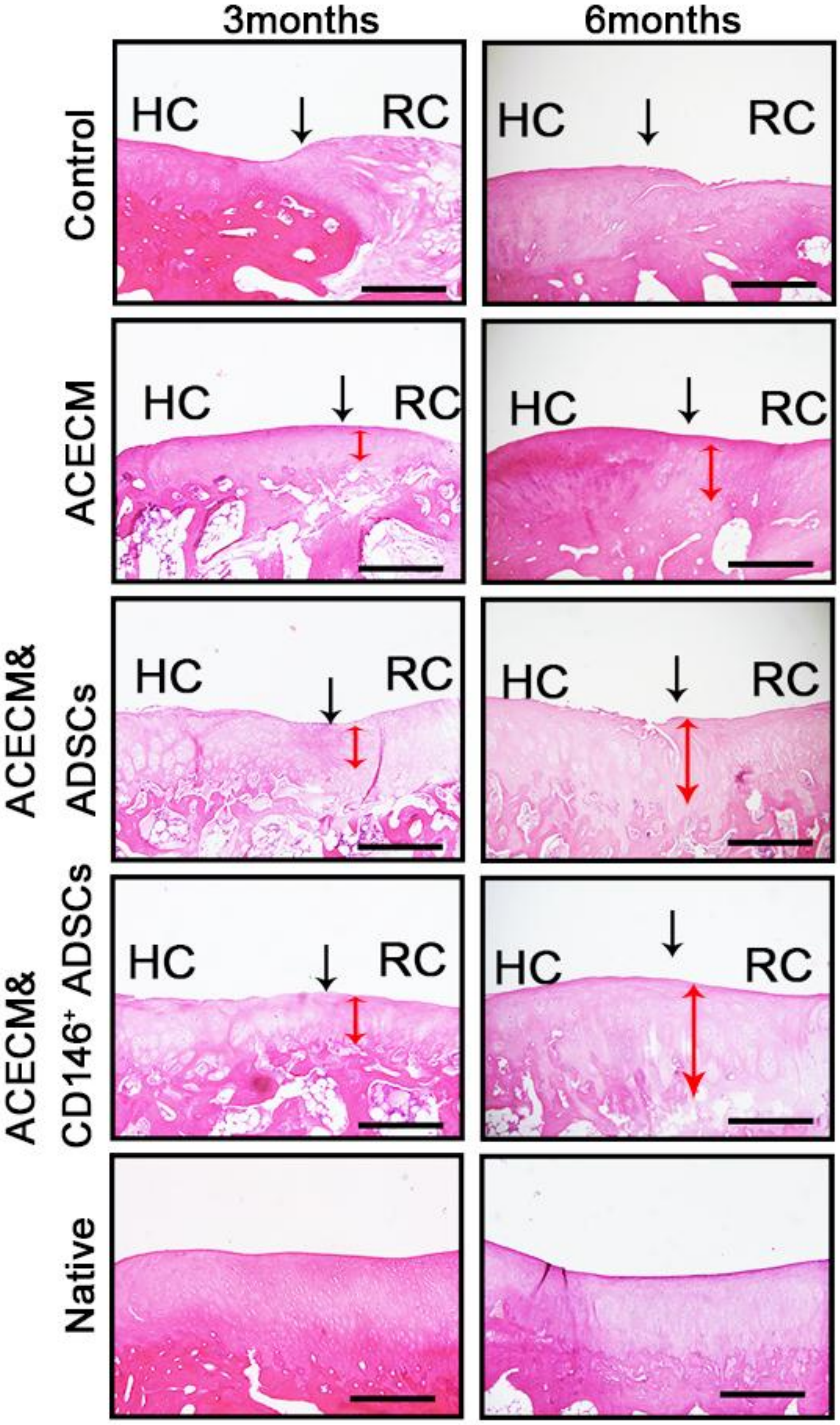

Figure S5. Histological analysis of cartilage defect after 3 and 6 months by H&E staining. The black solid arrows denote the repair interface. The red solid arrows denote the depth of the repaired cartilage. HC, host cartilage; RC, repaired cartilage. Scale bar: 200 μm.

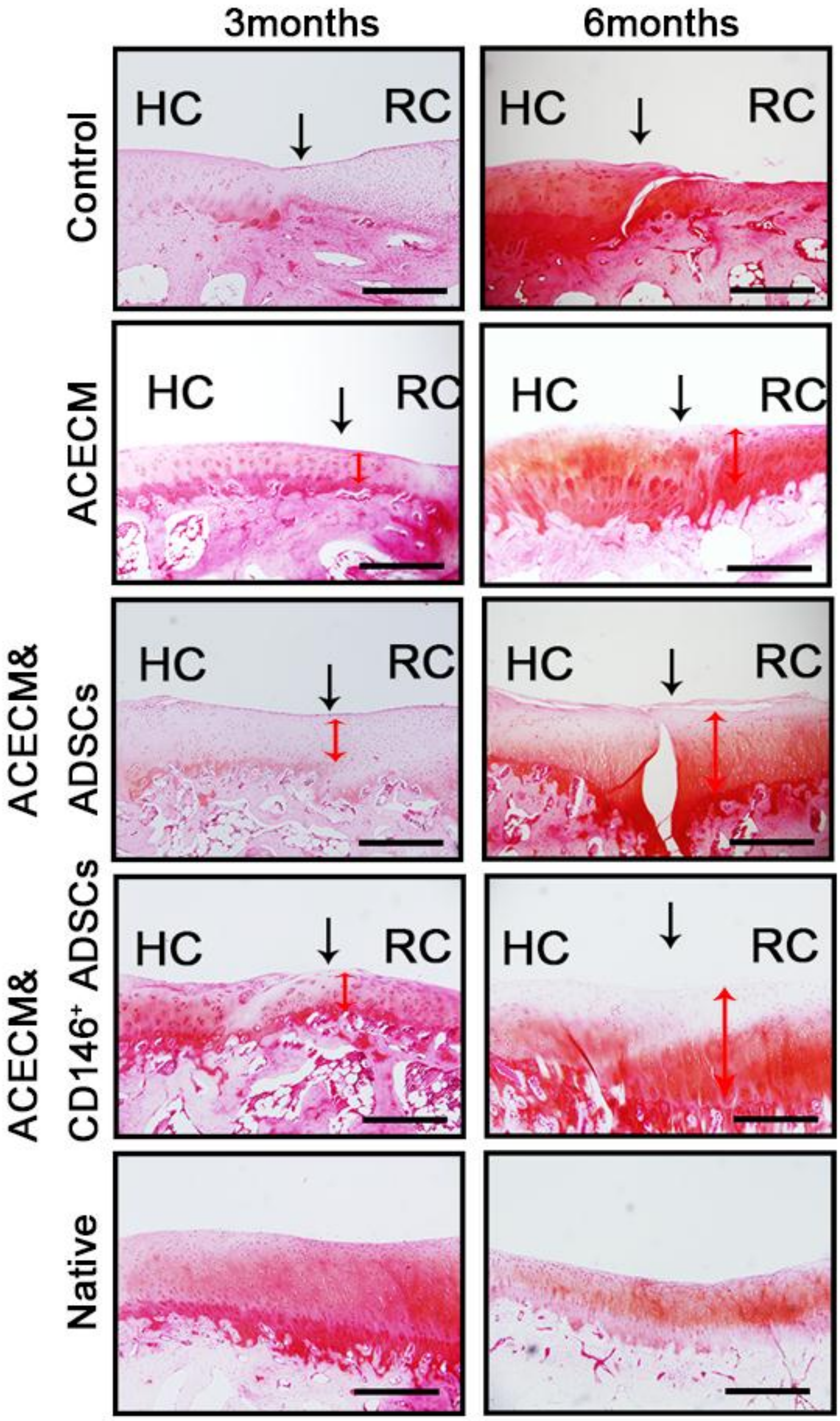

Figure S6. Histological analysis of cartilage defect after 3 and 6 months by Safranin O staining. The black solid arrows denote the repair interface. The red solid arrows denote the depth of the repaired cartilage. HC, host cartilage; RC, repaired cartilage. Scale bar: 200 μm

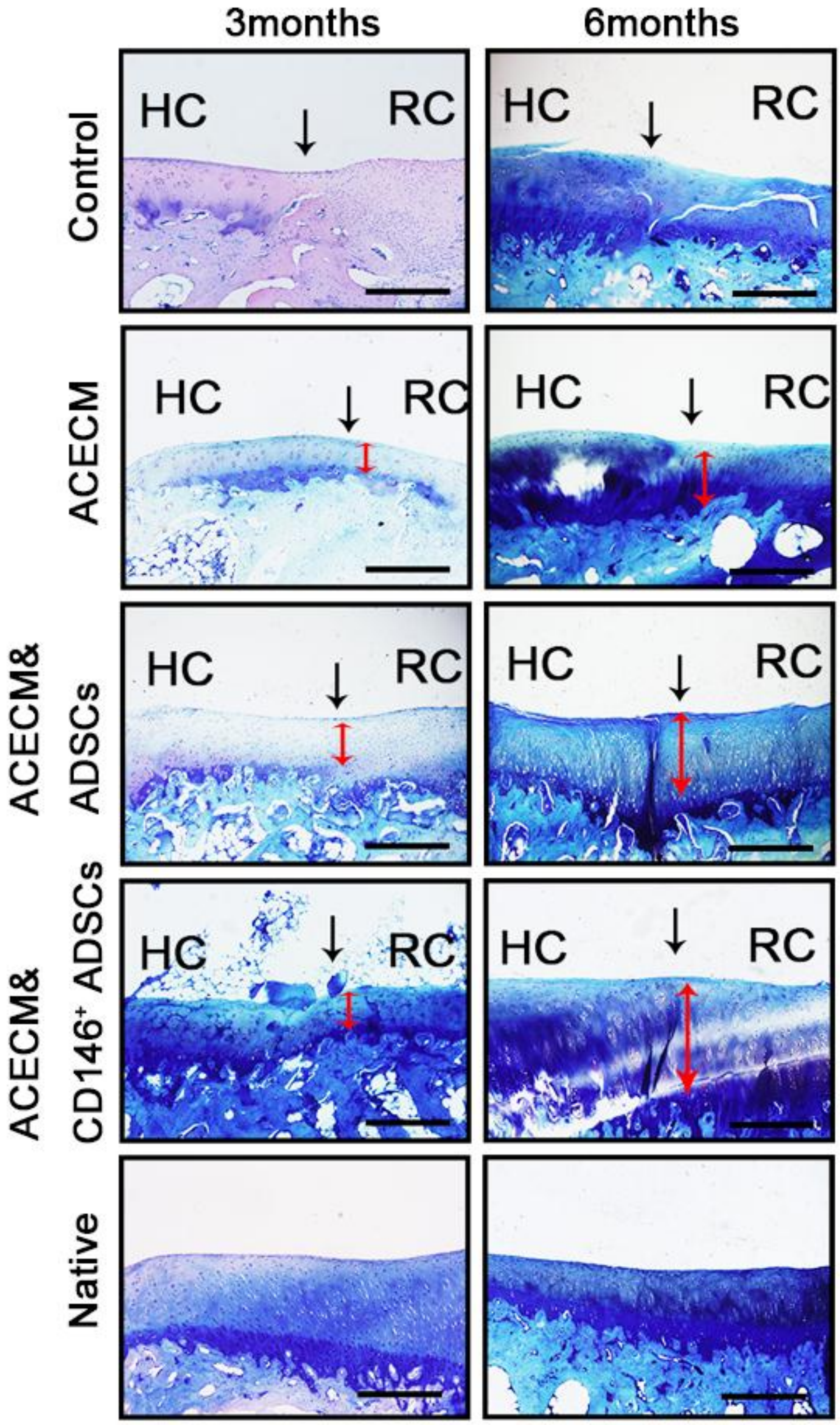

Figure S7. Histological analysis of cartilage defect after 3 and 6 months by Toluidine Blue staining. The black solid arrows denote the repair interface. The red solid arrows denote the depth of the repaired cartilage. HC, host cartilage; RC, repaired cartilage. Scale bar: 200 μm.

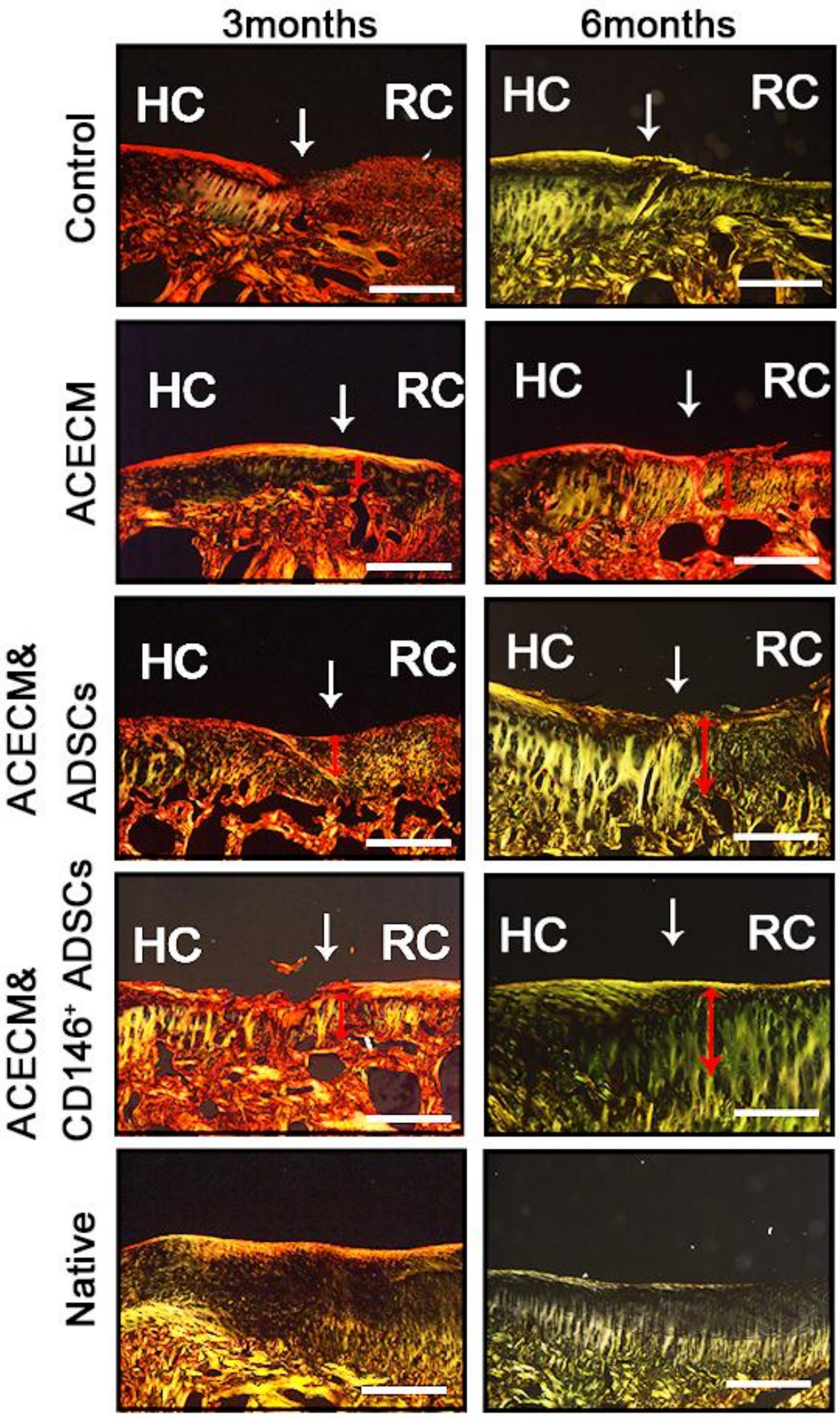

Figure S8. Histological analysis of cartilage defect after 3 and 6 months by Sirius Red staining. The black solid arrows denote the repair interface. The red solid arrows denote the depth of the repaired cartilage. HC, host cartilage; RC, repaired cartilage. Scale bar: 200 μm.

Table S1. List of Primers Used for RT-PCR Analysis

Gene	Forward primer	Reverse primer
CD80	GCTGGCTGGTCTTTCTCACT	GTGCCAGCTCTTCAACAGAA
CD40	CTCACCTCGCTATGGTTCGT	GGCACAAAGAACAGCACTGA
IdO1	GATGAAGAAGTGGGCTTTGC	CAGGCAGATGTTTAGCAATGA
PGE2	CCTTCTTCGAAAGTTTTGCC	ATGTGCAGTTGCCCTCTGTA
HGF	CGCTGGGAGTACTGTGCAAT	CCCTGTAGCCTTCTCCTTGA
IL-10	GTGGAGCAGGTGAAGAATGC	GCCACCCTGATGTCTCAGTT
ACTIN	ACCTTCTACAATGAGCTGCG	CCTGGATAGCAACGTACATGG
GAPDH	CAAGAAGGTGGTGAAGCAGG	GGTGTCGCTGTTGAAGTCAG

Table S2. Score System for MRI Evaluation

Criteria	Findings	Scores
**Repaired cartilage signal**	Isointense	2
	Hyperintense	1
	Hypointense	0
**Repaired lesion morphologic features**	Flush	2
	Proud	1
	Depressed	0
**Repaired cartilage fill**	Good (67%-100%)	2
	Moderate (34%-66%)	1
	Poor (0%-33%)	0
**Peripheral repaired cartilage integration**	No gap	2
	Small (gap of $\leqslant$2 mm)	1
	Large (gap of >2 mm)	0
**Subchondral edema**	None	3
	Mild (<1 cm2)	2
	Moderate (1-3 cm2)	1
	Severe (>3 cm2)	0
**Osseous overgrowth**	No	1
	Yes	0

Table S3. International Cartilage Repair Society Macroscopic Evaluation of Cartilage Repair

Cartilage repair assessment ICRS	Points
Degree of defect repair	
In level with surrounding cartilage	4
75% repair of defect depth	3
50% repair of defect depth	2
25% repair of defect depth	1
0% repair of defect depth	0
Integration to border zone	
Complete integration with surrounding cartilage	4
Demarcating border <1 mm	3
3/4th of graft integrated, 1/4th with a notable border >1 mm width	2
1/2 of graft integrated with surrounding cartilage, 1/2 with a notable border >1 mm	1
From no contact to 1/4th of graft integrated with surrounding cartilage	0
Macroscopic appearance	
Intact smooth surface	4
Fibrillated surface	3
Small, scattered fissures or cracks	2
Several, small or few but large fissures	1
Total degeneration of grafted area	0
Overall repair assessment	
Grade I: normal	12
Grade II: nearly normal	11–8
Grade III: abnormal	7–4
Grade IV: severely abnormal	3–1

ICRS, International Cartilage Repair Society.

Table S4. International Cartilage Repair Society Visual Histological Assessment Scale for Cartilage Repair

Feature	Points
I. Surface	
Smooth/continuous	3
Discontinuities/irregularities	0
II. Matrix	
Hyaline	3
Mixture: hyaline/fibrocartilage	2
Fibrocartilage	1
Fibrous tissue	0
III. Cell distribution	
Columnar	3
Mixed/columnar clusters	2
Clusters	1
Individual cells/disorganized	0
IV. Cell population viability	
Predominantly viable	3
Partially viable	1
<10% viable	0
V. Subchondral bone	
Normal	3
Increased remodeling	2
Bone necrosis/granulation tissue	1
Detached/fracture/callus at base	0
VI. Cartilage mineralization (calcified cartilage)	
Normal	3
Abnormal/inappropriate location	0
